# Supplementary material for: Confinement-enhanced valorization of contaminants in electrified hydrogenation membranes for water purification
Source: Nat Commun. 2025 Oct 27;16:9443. doi: 10.1038/s41467-025-64482-z (PMC12559343; doi:10.1038/s41467-025-64482-z)
Supplement: Supplementary file 1 — Supplementary Information [file 41467_2025_64482_MOESM1_ESM.pdf]

Supplementary Information

Confinement-enhanced valorization of contaminants in electrified hydrogenation membranes for water purification

Yuyang Kang, Zhenao Gu*, Wei Zhang, Baiwen Ma, Chenghai Lu, Chengzhi Hu, Jiuhui Qu

*Corresponding authors: Zhenao Gu
Email: zagu@rcees.ac.cn (Z.G.)

This file includes:

- Supplementary Methods
- Supplementary Figures 1 to 42
- Supplementary Tables 1 to 6
- Supplementary References

Supplementary Methods

Chemicals and Materials

Ruthenium (III) chloride ($RuCl_3$, 99.5%), trichloroacetic acid (TCAA, ≥99%), and dichloroacetic acid (DCAA, ≥99%) were purchased from Innochem. Sodium sulfate (Na_2SO_4, ≥99%), sodium hydroxide (NaOH, ≥96%), sodium nitrate ($NaNO_3$, ≥99%), sodium nitrite ($NaNO_2$, ≥99%), ammonium sulfate (≥99%), acetic acid (AA, ≥99.8%), formic acid (HPLC grade), cyclohexanol (≥99.5%), cyclohexanone (≥99.5%), Nessler's reagent, sulfamic acid (≥99.5%), potassium sodium tartrate (≥99%), hydrochloric acid (36%–38%), p-aminobenzenesulfonamide (≥99.8%). phosphoric acid (≥85%), N-(1-Naphthyl) ethylenediamine dihydrochloride (≥97%), copper (II) sulfate pentahydrate (≥99%), bromothymol blue, and phenol (≥99%) were purchased from Sinopharm (China). Chloroacetic acid (MCAA, 99%) was purchased from Alfa Aesar. The compound 2′,7′-Bis(2-carboxyethyl)-5 (6)- carboxyfluorescein acetoxymethyl ester (BCECF-AM) was purchased from Beyotime (China), and 5,5-dimethyl-1-pyrroline N-oxide (DMPO, ⩾97%) were purchased from Ark Pharm. Acetonitrile (HPLC grade), and methanol (HPLC grade) were purchased from Fisher Scientific. All chemicals were used as received. Ti substrate was purchased from Nanjing Shinkai Filter Co., Ltd (China). All solutions were prepared using deionized water purified by a Milli-Q Plus system (Millipore).

Analysis Methods

Determination of NO_3^-: For NO_3^- measurement, 1 mL of the sample was collected and diluted to 5 mL using electrolyte solution. Subsequently, 100 μL of 1 M HCl and 10 μL of 0.8 wt% sulfamic acid solution were added to the diluted electrolyte. After 15 min, the adsorption spectrum at the wavelength of 220 nm and 275 nm was measured using a UV-Vis spectrometer. A standard curve was obtained from the corresponding absorbance for the concentration of NO_3^- standard solution.

Determination of NO_2^-: For NO_2^- measurement, 5 mL of the sample was used without dilution. Subsequently, 100 μL of the color reagent (0.2 g of N-(1-Naphthyl) ethylenediamine dihydrochloride and 4 g of p-aminobenzenesulfonamide in 10 mL of phosphoric acid (1.70 g mL^{-1})) was added to the diluted sample. After 20 min, the adsorption spectrum was measured at the wavelength of 540 nm. The standard curve was obtained using a series of NO_2^--N standard solutions.

Determination of NH_4^+: For NH_4^+ measurement, 0.2 mL of the sample was taken and diluted to 2 mL with ultrapure water. Nessler's reagent and potassium sodium tartrate solution (500 g L^{-1})

were employed as a color reagent for NH_4^+ and a masking agent for metal ions, respectively. After adding 60 μL Nessler's reagent and 40 μL potassium sodium tartrate solution, the mixture was left to stand for 30 min and the adsorption spectrum at the wavelength of 420 nm was measured. The standard curve was obtained by using a series of NH_4^+ standard solutions.

Electrical Energy Consumption

The total energy consumption was calculated as the sum of electrical energy ($E_{\text{electrical}}$, kWh g-N^{-1}) and pumping energy (E_{pumping}) normalized per 80% removal of nitrate.

The nominal hydraulic residence time (HRT_{geom}) required to achieve an 80% removal rate was calculated using Supplementary Equation (1),

$$\text{HRT}_{\text{geom,80\%}} = \frac{\ln(5)}{k_{\text{obs}}} \quad (1)$$

The $E_{\text{electrical}}$ value was calculated according to Supplementary Equation (2)[1],

$$E_{\text{electrical}} = 10^{-3} \times \frac{V_{\text{cell}} I}{QC} \quad (2)$$

where V_{cell} is the cell potential (V), I is the current used in the experiment (i.e., 2.54 cm^2 × 39.4 mA cm^{-2} × 10^{-3} = 0.1 A), Q is the volumetric flow rate at which 80% nitrate removal was achieved (m^3 h^{-1}), C is the concentration of nitrate removed (80% of the initial concentration) (g-N m^{-3}).

The Q value was calculated by Supplementary Equation (3),

$$Q = 3600 \times \frac{V_{\text{geom}}}{\text{HRT}_{\text{geom,80\%}}} \quad (3)$$

where V_{geom} is the geometric volume of the electrified membrane (EM) (7.6×10^{-7} m^3).

The E_{pumping} value was calculated by Supplementary Equation (4)[1],

$$E_{\text{pumping}} = 3.6 \times 10^{-5} \times \frac{\rho g \Delta P}{\eta C} \quad (4)$$

where ρ is the density of water (997 kg m^{-3}), g is the gravitational constant (9.81 m s^{-2}), ΔP is the transmembrane pressure at a given flux (bar), and η is the pump efficiency (assumed as 0.7).

Multiphysics Simulation

The multiphysics analysis was simulated using COMSOL Multiphysics 5.3a. The pore diameter was simplified to the modal pore size observed in EM experiments, ignoring the actual pore size distribution. Accordingly, EM channels with diameters of 3.8, 7.0, 24.0, 58.0, and 80.0 μm were modeled, respectively. Outlet and inlet were connected to both ends of the channel, respectively. The distance between the inlet and outlet is 3 mm, consistent with the thickness of EM. All channel models were assumed to have the same tortuosity (tortuosity = 1.4[2]) and were simplified as regular cylinders. The channel was modeled with a certain curvature to simulate the tortuosity inside the EMs[2].

The 3D geometry "CFD" module was used to solve the flow velocity and streamline distribution in the simulated pores. The range of HRT_{geom} in the simulation (3–182 s) was consistent with the experiment (3–182 s). The Reynolds number (*Re*) is defined by Supplementary Equation (5)[3,4],

$$Re = \frac{\rho U d_{\mathrm{p}}}{\mu} \tag{5}$$

where U is the flow velocity (m s^{-1}), d_{p} is the diameter of the pore (m), and μ is the dynamic viscosity (kg m^{-1} s^{-1}). Since the *Re* remains below 1 even at the highest flow velocities on EMs (Supplementary Table 5), the flow in the microchannels was confirmed to be laminar. The solution's inflow/outflow was modeled as steady-state flow (transient velocity fluctuations were not accounted for).

The properties of the electrode and electrolyte are assumed homogeneous throughout the pore. The electrode was treated as highly conductive, neglecting internal IR drop, and therefore it is not affected by the limiting current density (i.e., the local maximum current density in the electrode). The "transport of diluted species" module was used to solve the concentration distribution. The mathematical model of the mass transfer process includes Fick's law, the convection process, and the reaction process. A 100 mM Na_2SO_4 electrolyte was used in the experiment, allowing us to ignore the electrostatic migration of NO_3^- and NH_4^+, thus simplifying the model[5]. The disturbance of gas evolution on flow and mass transfer was neglected. The channels were assumed to be fully electrolyte-filled, with no gas blockage. The convection–diffusion equation for each species was given by Supplementary Equation (6)[6],

$$R_{\mathrm{i}} = -\nabla \cdot [D_{\mathrm{i}} \nabla C_{\mathrm{i}} + C_{\mathrm{i}} \mathrm{u}] \tag{6}$$

where C_i is the concentration of species i, R_i is the reaction flux of species i, and D_i is the diffusion coefficient of species i.

As the current density is influenced by the depth of pore[7,8], the "secondary current distribution" module was used to investigate the current distribution in the flow-through system. The exchange current density was determined by Supplementary Equation (7),

$$i_0 = Fk^0 C_\mathrm{O}^{*(1-\alpha)} C_\mathrm{R}^{*\alpha} \quad (7)$$

where i_0 is the exchange current density (A m^{-2}), F is the Faraday constant (C mol^{-1}), k^0 is the standard heterogeneous rate constant, α is the charge transfer coefficient, C_O^*, and C_R^* are the bulk concentration of oxidized species, and reduced species, respectively. According to recent studies, the electrocatalytic reaction of Ru is not significantly affected by pH[9,10] . Furthermore, all experiments were conducted under the same conditions of pH = 7 and a current density of 39.3 mA cm^{-2}. Therefore, the effect of pH was not considered in the model.

The overpotential (η) was calculated by Supplementary Equation (8)[11],

$$\eta = \varphi_\mathrm{m} - \Delta\varphi_\mathrm{s} - \varphi_\mathrm{e} \quad (8)$$

where φ_m is the electrode potential (V), $\Delta\varphi_\mathrm{s}$ is the solution potential drop (V), and φ_e is the equilibrium potential (V).

The current-overpotential equation was used to solve for the current distribution (Supplementary Equation (9))[12],

$$i = i_0 \left[\frac{C_\mathrm{R}}{C_\mathrm{R}^*} e^{\frac{\alpha F \eta}{RT}} - \frac{C_\mathrm{O}}{C_\mathrm{O}^*} e^{\frac{-(1-\alpha)\eta F}{RT}} \right] \quad (9)$$

where i is the local current density (A m^{-2}).The applied current (i_{app}, A) of one channel was determined according to the porosity and current in the experiment (Supplementary Equation (10)),

$$i_\mathrm{app} = I \frac{S}{pA} \quad (10)$$

where I is the current in the experiment (0.1 A unless otherwise mentioned), p is the porosity of EM, A is the geometry surface area of the electrode (2.54×10^{-4} m^2), and S (m^2) is the cross-section area of the simulated pore along the axial direction.

The outer surface of the channel was also designated as electroactive to simulate the actual situation. The equivalent diameter (d^*) of this region was determined according to the porosity and the pore diameter (Supplementary Equation (11)),

$$d^* = \frac{d}{\sqrt{p}} \tag{11}$$

where d is the diameter of the simulated channel (m).

Two reactions were defined on the electrode surface:

No.	Reactions	Standard Electrode Potential	Exchange Current Density	Transfer Coefficient
1	$2H_2O + 2e^- \rightarrow H_2 + 2OH^-$	E_H	i_H	α_H
2	$NO_3^- + 8e^- + 6H_2O \rightarrow NH_3 + 9OH^-$	E_{NH}	i_{NH}	α_{NO}

Only the two aforementioned reactions are considered on the cathode surface, neglecting the accumulation of intermediate products.

All parameters and values used in the simulation are shown in Supplementary Table 6. When the simulation was performed at different parameter values (e.g., pore size and electrolyte conductivity), all other parameters remained unchanged.

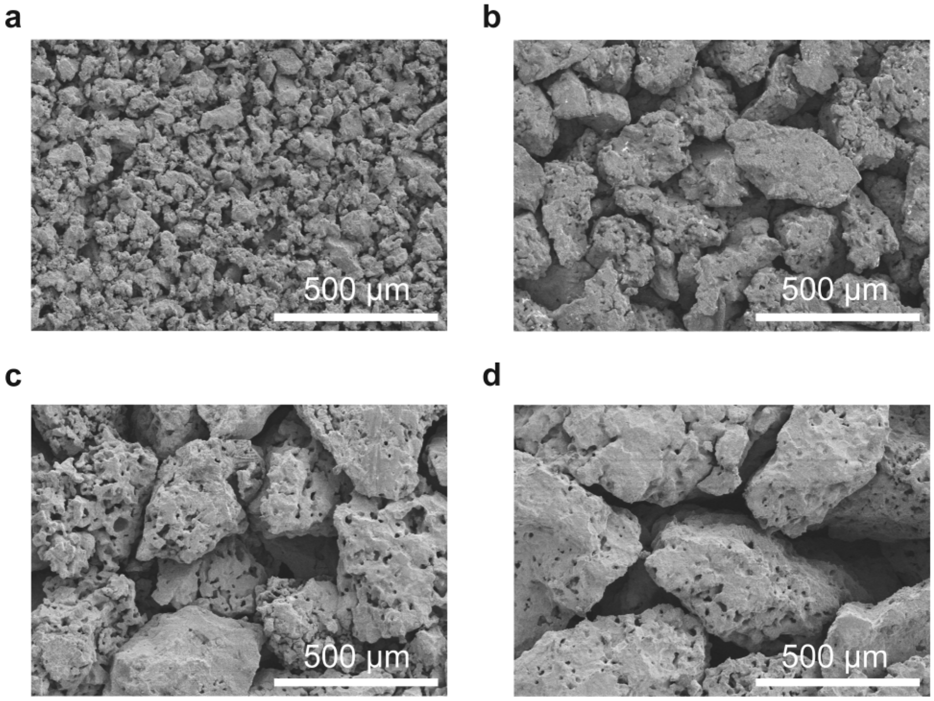

Supplementary Fig. 1. Macroscopic pores in electrified membranes (EMs). SEM images (top view) of (**a**) EM_7, (**b**) EM_{24}, (**c**) EM_{58}, and (**d**) EM_{80}.

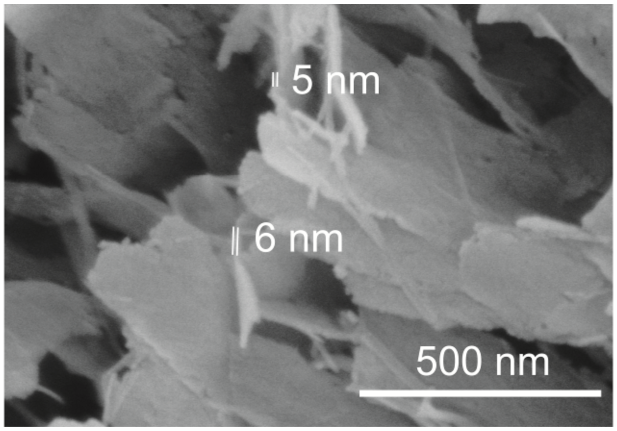

Supplementary Fig. 2. SEM image of the nanosheets on EMs.

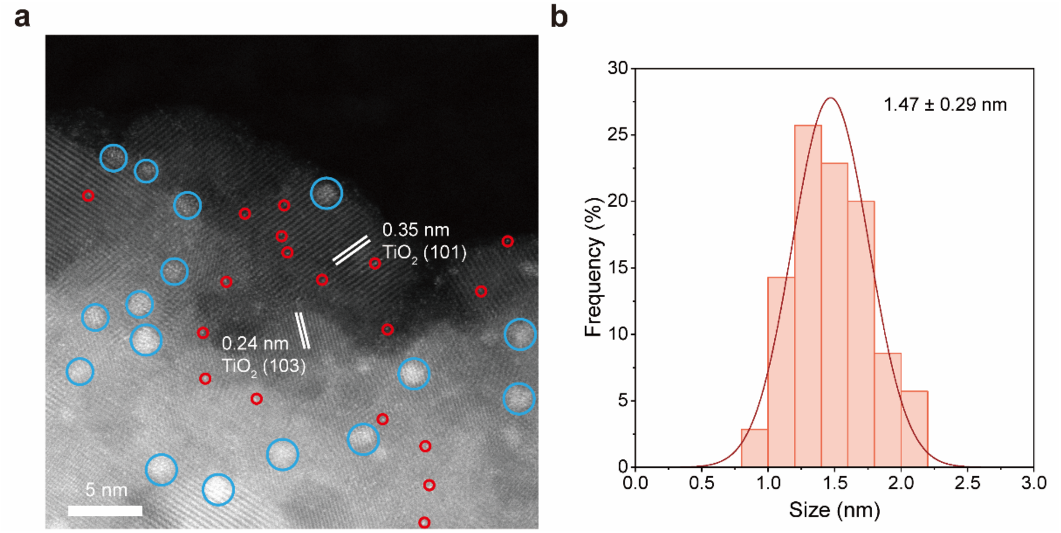

Supplementary Fig. 3. Ru species on the nanosheets of EMs. **a** High-angle annular dark-field imaging scanning transmission electron microscopy (HAADF-STEM) image of the Ru/TiO_2 EMs, where some of the Ru nanoclusters (NCs) and Ru single-atoms (SAs) are highlighted by blue and red circles, respectively, and (**b**) corresponding particle size histogram.

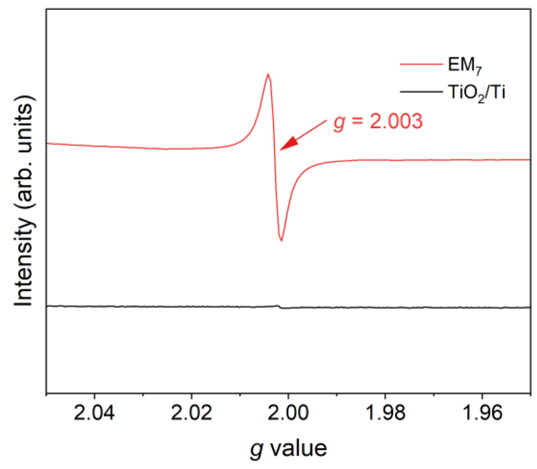

Supplementary Fig. 4. Electron spin resonance spectroscopy (ESR) spectra of EM_7 and TiO_2/Ti substrate. The signal at g = 2.003 is a typical sign of oxygen vacancies[13].

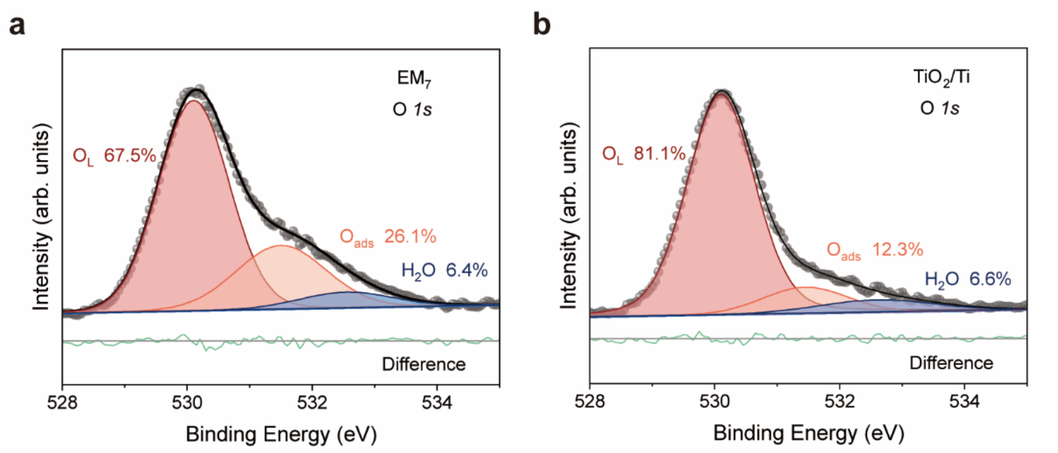

Supplementary Fig. 5. X-ray photoelectron spectroscopy (XPS) spectra of EM_7 and TiO_2/Ti substrate. **a** O *1s* XPS spectra of EM_7. **b** O *1s* XPS spectra of TiO_2/Ti substrate. The peaks at 530.3, 531.5, and 532.6 eV were attributed to lattice oxygen (O_L), adsorbed oxygen (O_{ads}), and surface oxygen (H_2O), respectively[14,15]. The increase of the O_{ads} demonstrates the existence of oxygen vacancies accompanied by localized electron richness[16].

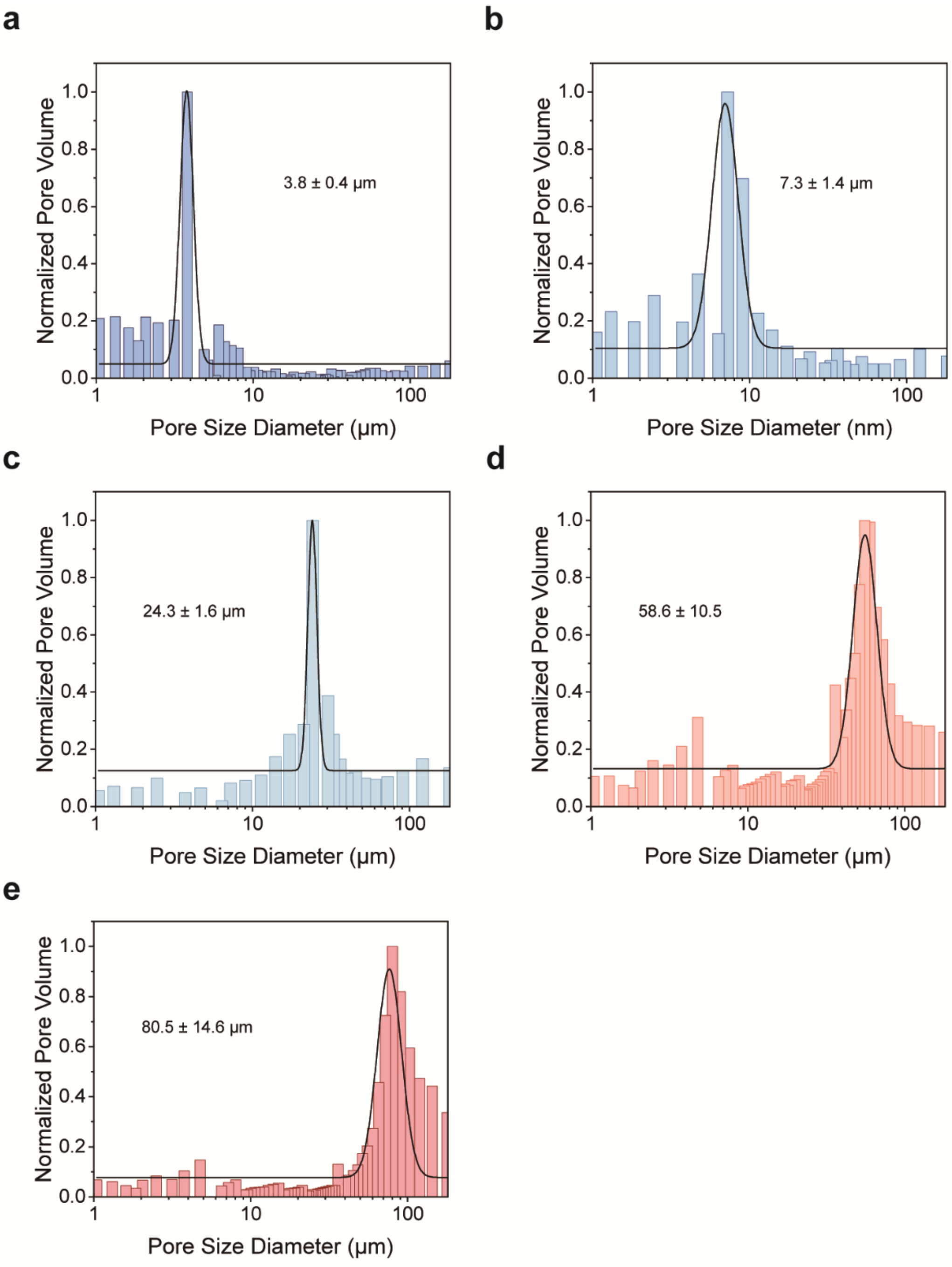

Supplementary Fig. 6. Log-normal fitting curves representing the pore size distributions. The individual panels correspond to distributions for (**a**) EM$_4$, (**b**) EM$_7$, (**c**) EM$_{24}$, (**d**) EM$_{58}$, and (**e**) EM$_{80}$.

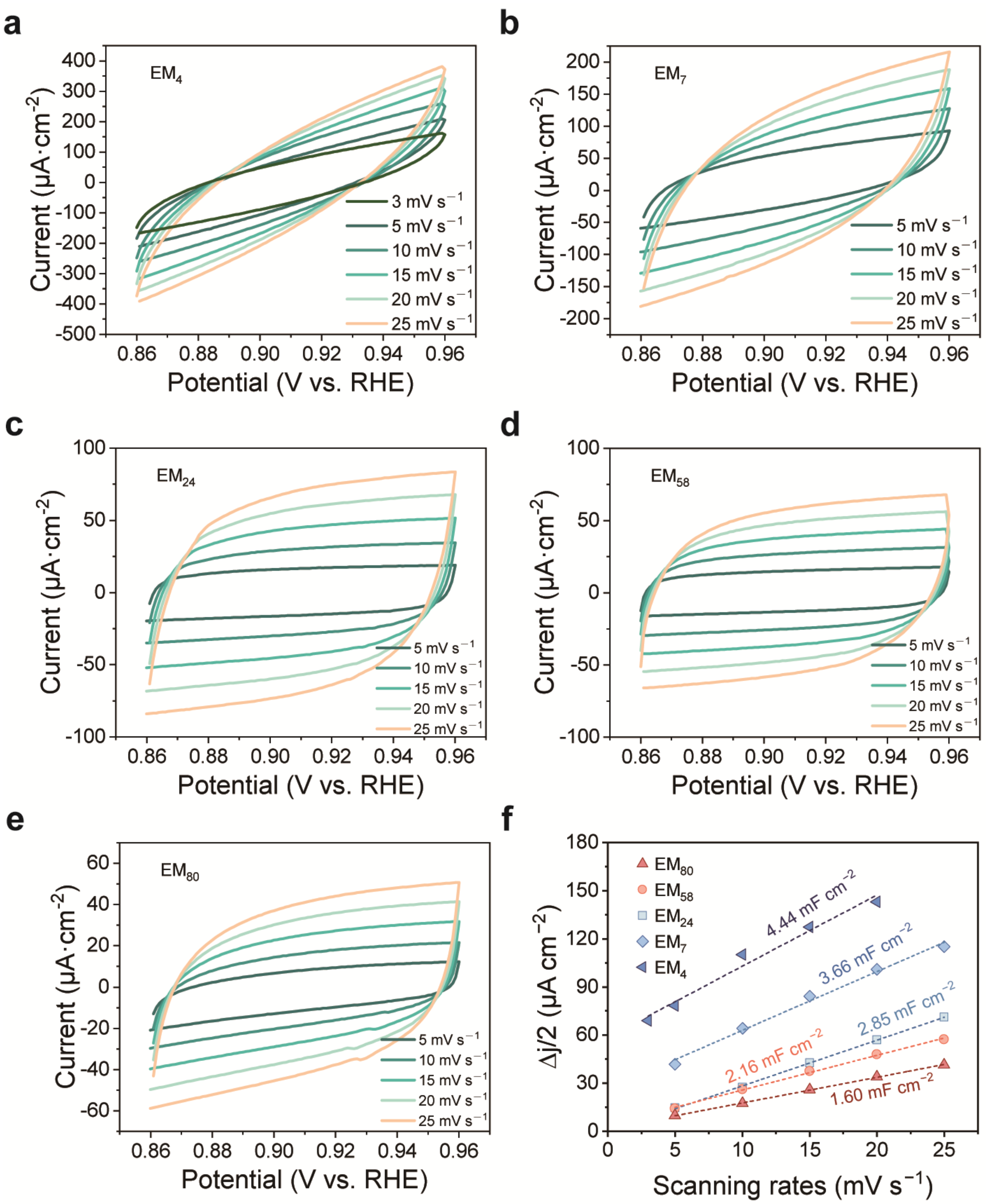

Supplementary Fig. 7. The cyclic voltammetry (CV) curves of EMs with different pore sizes and the corresponding electrical double-layer capacitance (C_{dl}). CV curves of (**a**) EM_4, (**b**) EM_7, (**c**) EM_{24}, (**d**) EM_{58}, (**e**) EM_{80}, and (**f**) C_{dl} by plotting current variation against the scan rate to fit a linear regression. C_{dl} values were measured at the potential of 0.91 V vs. RHE. Electrolyte: 0.1 M Na_2SO_4.

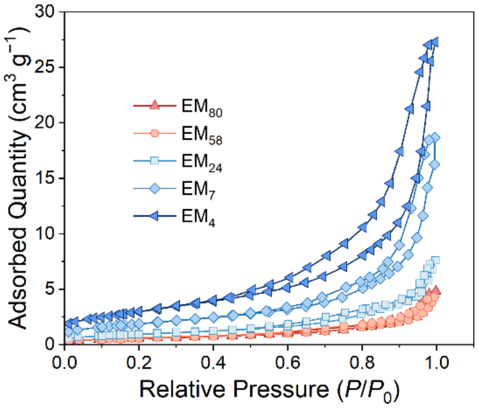

Supplementary Fig. 8. Nitrogen adsorption/desorption isotherms of EMs with different pore sizes.

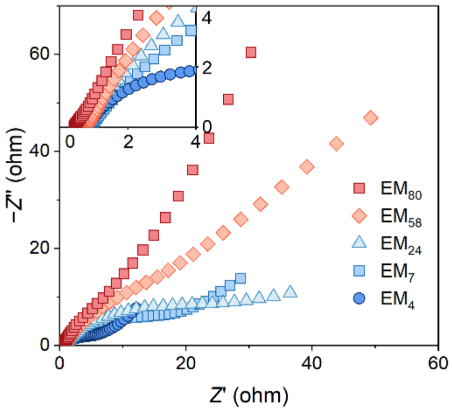

Supplementary Fig. 9. Electrochemical impedance spectroscopy of EMs (inset: magnified view illustrating the x-intercept).

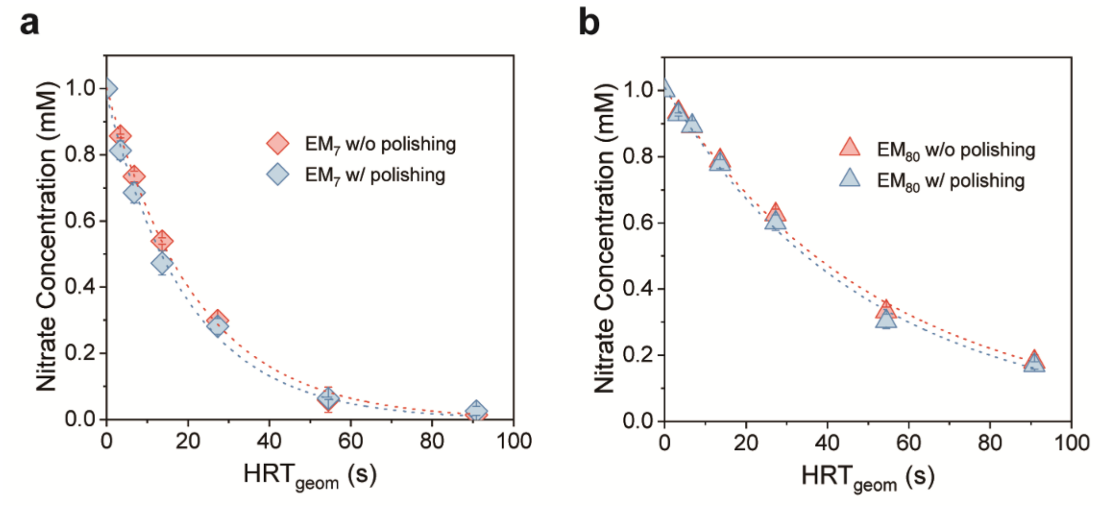

Supplementary Fig. 10. Nitrate removal performance of EMs with surface polishing. Panels correspond to results on (**a**) EM_7 and (**b**) EM_{80}. The dotted lines represent the pseudo-first-order kinetic regression curves ($R^2 > 0.99$). Error bars represent the standard deviation from at least two independent tests. Electrolyte: 0.1 M Na_2SO_4.

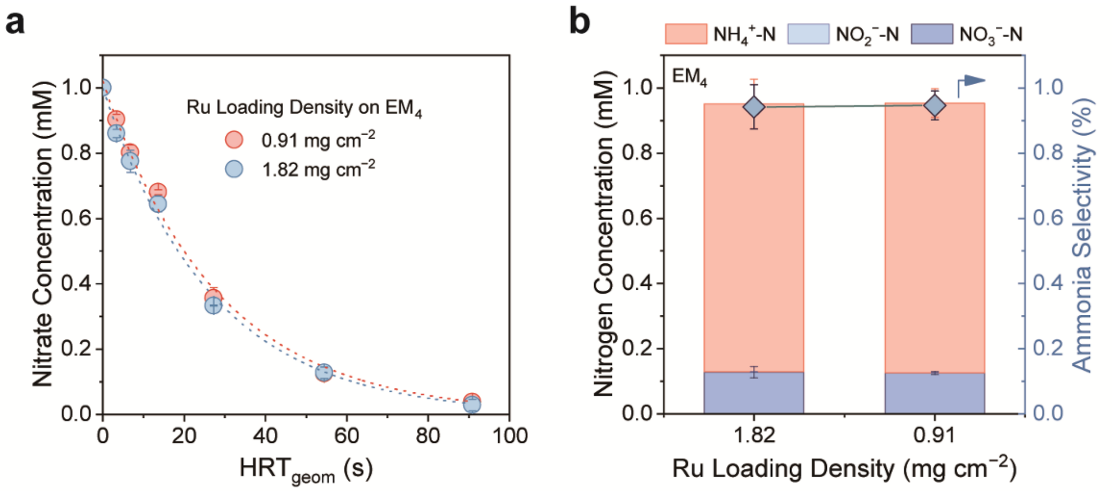

Supplementary Fig. 11. The nitrate removal performance on EM_4 with different Ru loading. (**a**) Removal of nitrate on EM_4 with different Ru loading. (**b**) Distribution of nitrogen species in the permeate (left axis) and NH_4^+ selectivity (right axis) at a hydraulic residence time (HRT_{geom}) of 55 s. The dotted lines represent the pseudo-first-order kinetic regression curves ($R^2 > 0.99$). Error bars represent the standard deviation from at least two independent tests. Electrolyte: 0.1 M Na_2SO_4.

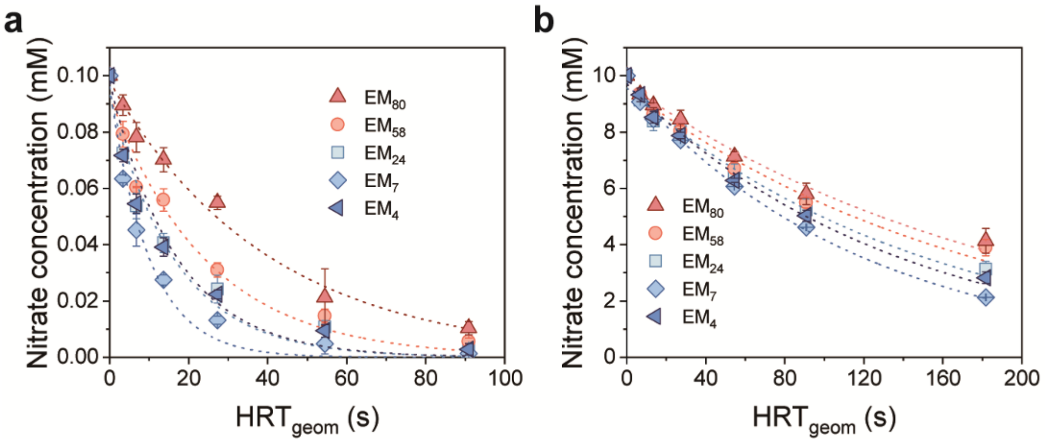

Supplementary Fig. 12. Nitrate removal performance on EMs at different nitrate concentrations. Panels correspond to results at concentrations of (**a**) 0.1 mM and (**b**) 10 mM. The dotted lines represent the pseudo-first-order kinetic regression curves (R^2 > 0.96). Error bars represent the standard deviation from at least two independent tests. Electrolyte: 0.1 M Na_2SO_4.

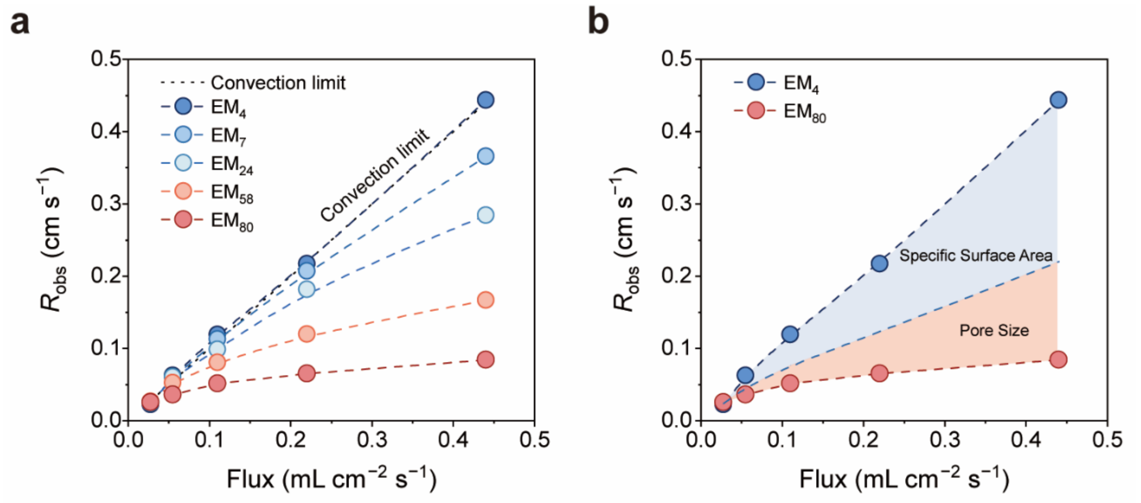

Supplementary Fig. 13. Mass transfer rate on EMs. (**a**) Observed mass transfer rate (R_{obs}) on EMs. (**b**) Comparison of R_{obs} between EM_4 and EM_{80}. Highlighted areas correspond to the contribution of surface area and pore size. Since EM_4's ECSA is 2.8 times that of EM_{80}, the orange region represents the maximum contribution (2.8×) from the specific surface area to mass transport, while the blue region reflects the minimal contribution from pore structure. Experiments were conducted at 0.26 V_{RHE} in an electrolyte containing 0.1mM Cu^{2+}, and 0.1M Na_2SO_4.

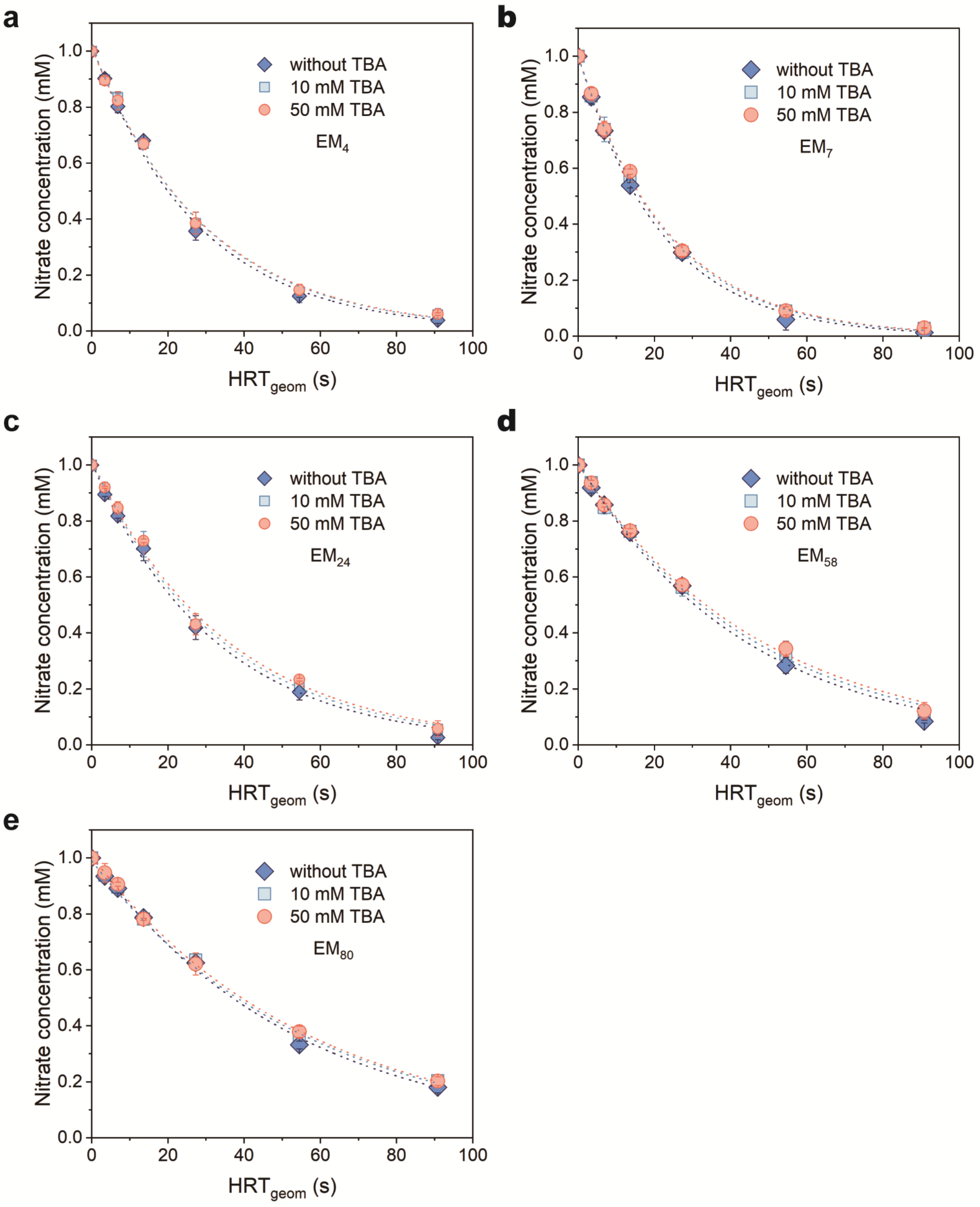

Supplementary Fig. 14. Removal of nitrate as a function of time in the presence of different concentrations of *tert*-butyl alcohol (TBA).The panels depict the results for (**a**) EM_4, (**b**) EM_7, (**c**) EM_{24}, (**d**) EM_{58}, and (**e**) EM_{80}. The dotted lines represent the pseudo-first-order kinetic regression curves ($R^2 > 0.99$). Error bars represent the standard deviation from at least two independent tests. Electrolyte: 0.1 M Na_2SO_4.

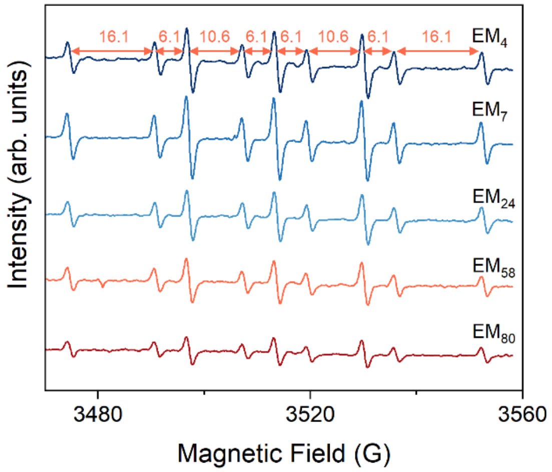

Supplementary Fig. 15. DMPO spin trapping ESR spectra on EMs. Electrolyte: 0.1 M Na_2SO_4.

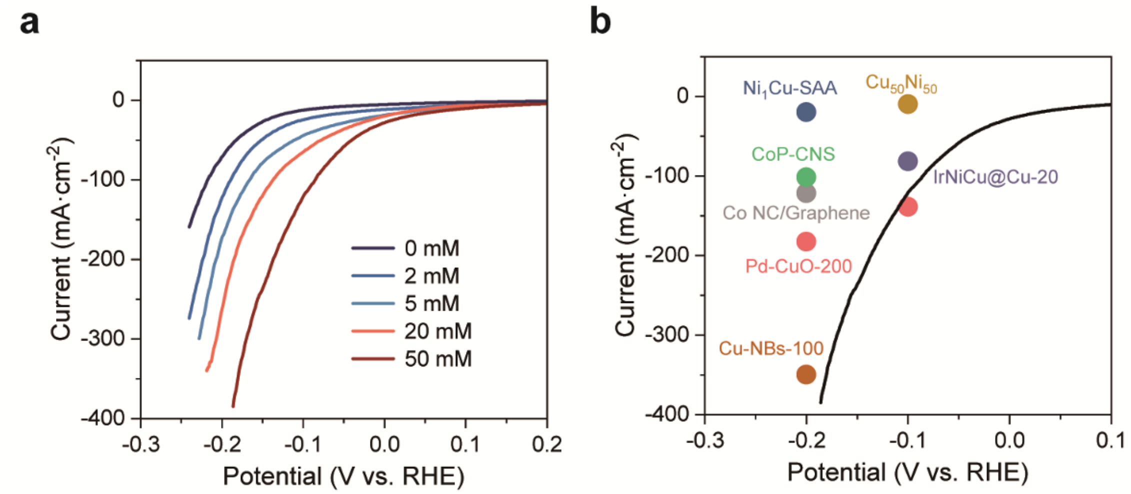

Supplementary Fig. 16. Nitrate reduction current on EM_7. (**a**) Linear sweep voltammetry (LSV) curves of EM_7 in the presence of different concentrations of nitrate. (**b**) Comparison of the nitrate reduction current on EM_7 with that of other electrocatalysts. Details included in Supplementary Table 3. Electrolyte: 0.1 M Na_2SO_4 (pH = 7).

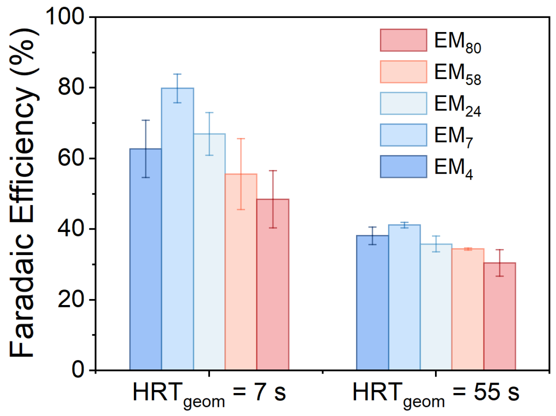

Supplementary Fig. 17. Faradaic efficiencies (FE) for nitrate reduction on EMs at HRT_{geom} values of 3, and 27 s. Error bars represent the standard deviation from at least two independent tests. Nitrate concentration: 10 mM. Electrolyte: 0.1 M Na_2SO_4 (pH = 7).

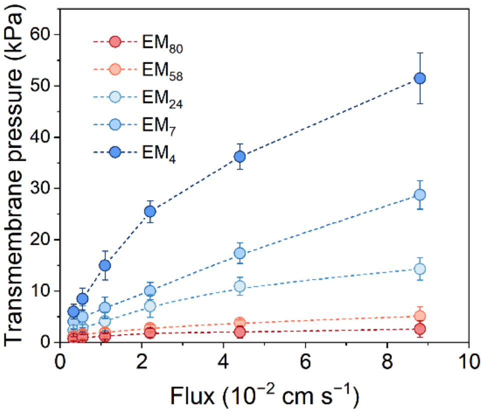

Supplementary Fig. 18. Transmembrane pressure as a function of flux. Error bars represent the standard deviation from at least two independent tests.

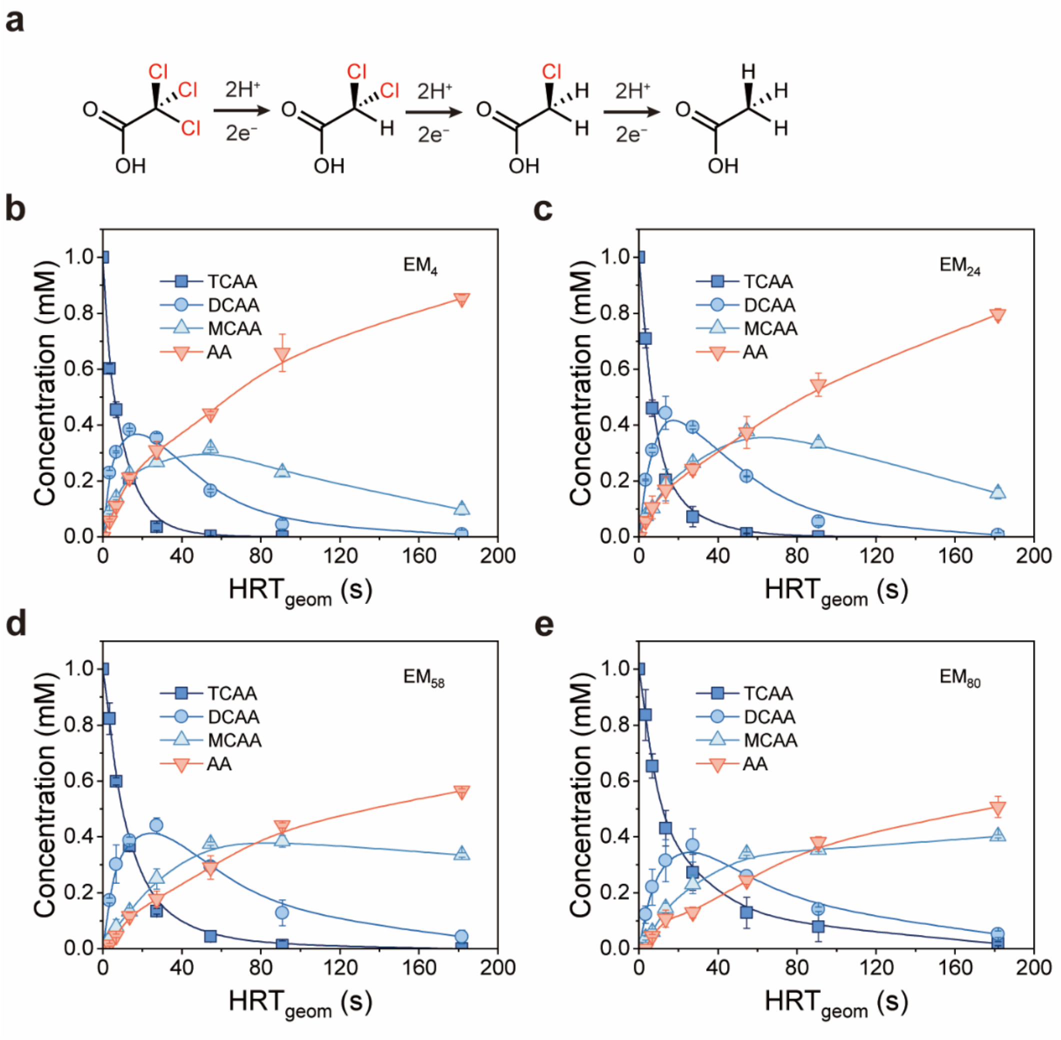

Supplementary Fig. 19. Concentration evolution of TCAA and its dechlorination products. (**a**) Proposed TCAA ECH reaction pathways on EMs. Other panels correspond to results on (**b**) EM_4, (**c**) EM_{24}, (**d**) EM_{58}, and (**e**) EM_{80}. The solid lines depict the schematic variation in concentration. Error bars represent the standard deviation from at least two independent tests. Electrolyte: 0.1 M Na_2SO_4 (pH = 7).

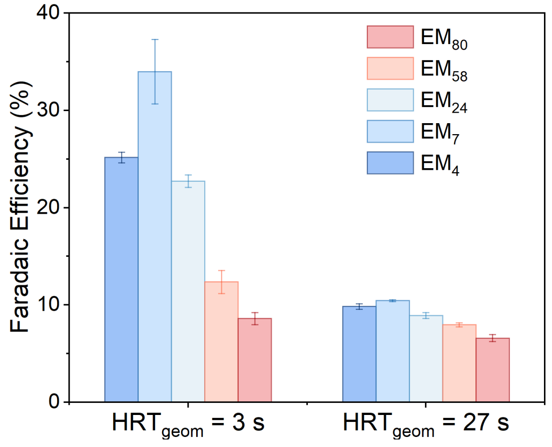

Supplementary Fig. 20. FEs for TCAA conversion on EMs at HRT_{geom} values of 3, and 27 s. TCAA concentration: 1 mM. Error bars represent the standard deviation from at least two independent tests. Electrolyte: 0.1 M Na_2SO_4 (pH = 7).

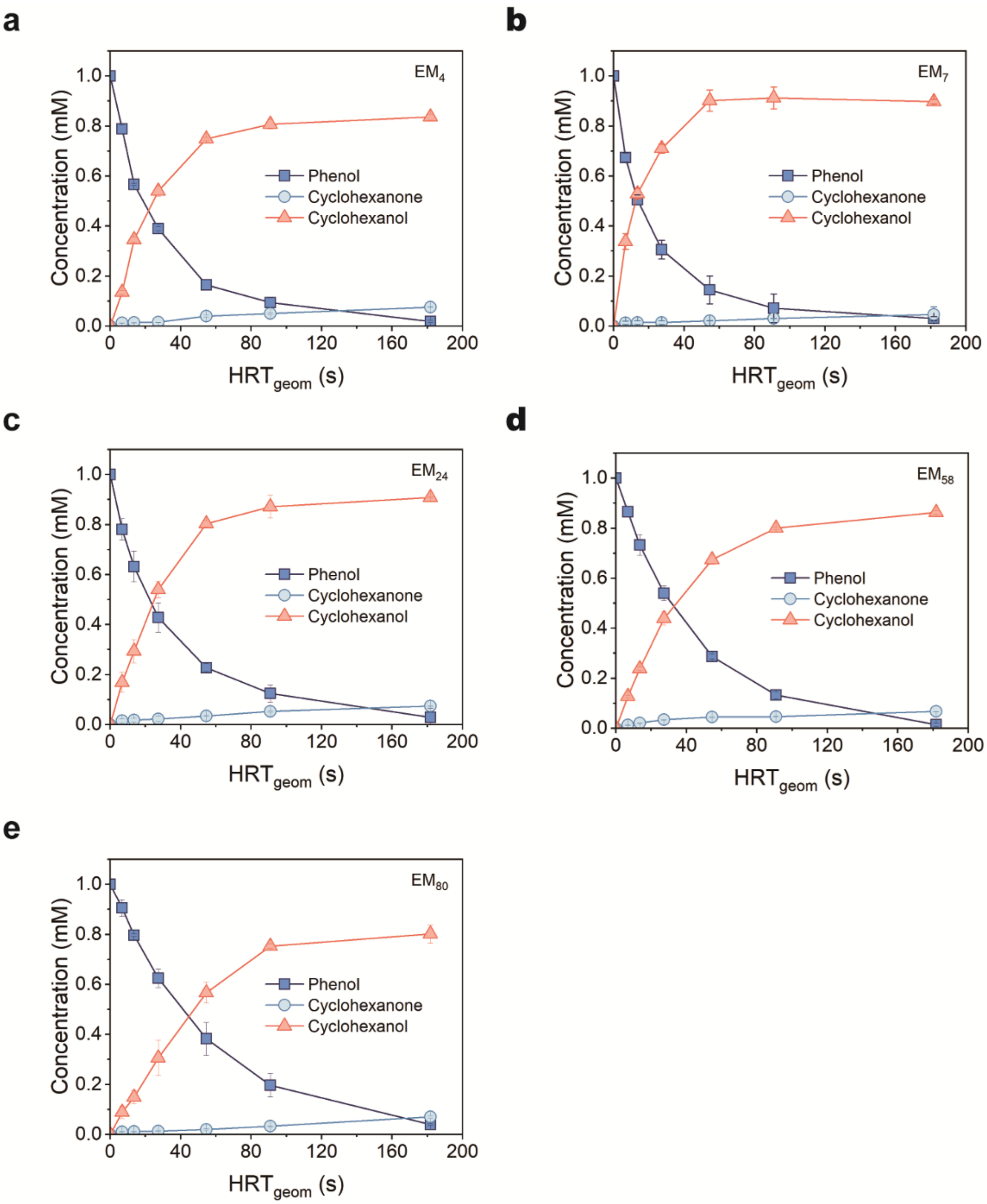

Supplementary Fig. 21. Concentration evolution of phenol and its reduction products. Panels correspond to results on (**a**) EM$_4$, (**b**) EM$_7$, (**c**) EM$_{24}$, (**d**) EM$_{58}$, and (**e**) EM$_{80}$. Error bars represent the standard deviation from at least two independent tests. Electrolyte: 0.1 M Na_2SO_4 (pH = 7).

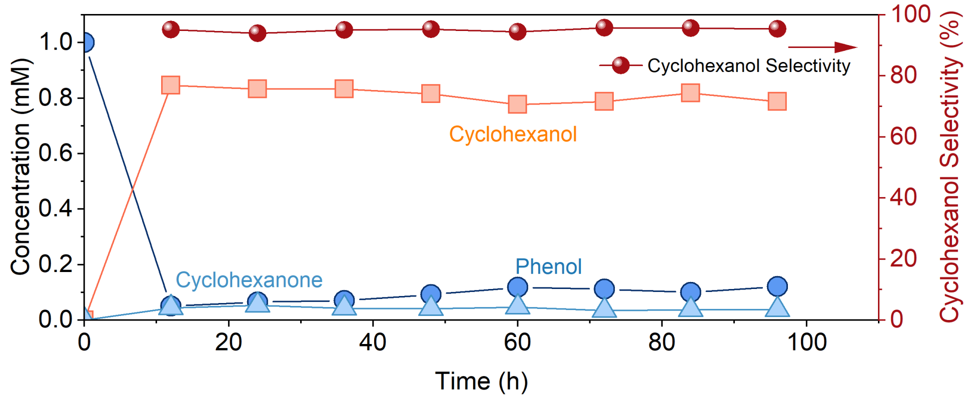

Supplementary Fig. 22. Long-term nitrate reduction stability test of EM_7 with 1 mM nitrate at an HRT_{geom} of 91 s. Current density: 39.3 mA cm^{-2}, reaction conditions: 0.1 M Na_2SO_4, pH = 7.

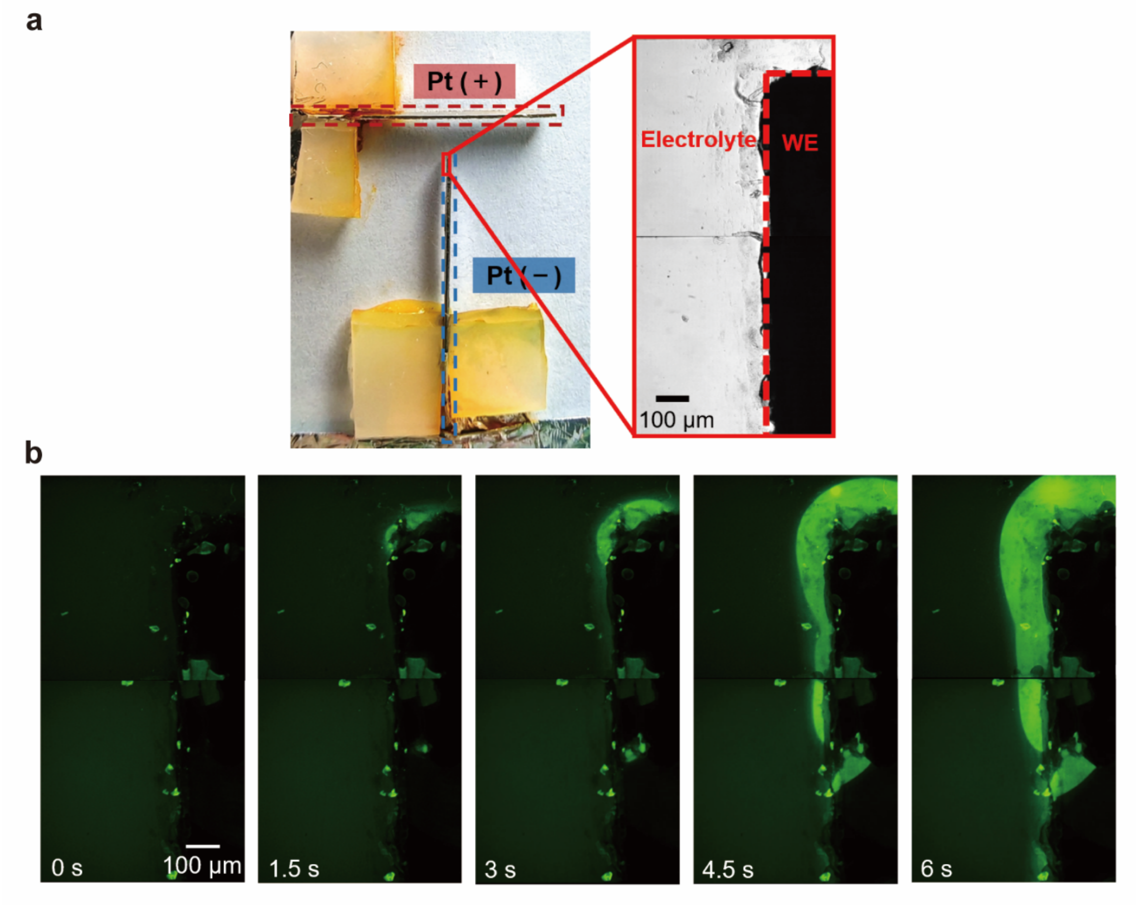

Supplementary Fig. 23. Fluorescence observation of current density distribution on Pt electrode surface. (**a**) Photograph of electrode configuration. (**b**) Evolution of pH distribution at electrode interface. The working electrode (WE) was positioned perpendicular to the counter electrode to simulate the EM. Due to instrument limitations, the upper and lower parts of the electrode were recorded in two separate repeat experiments. BCECF-AM was employed as OH^- probe in solution. Current: 0.5 mA, electrolyte: 0.02 M Na_2SO_4.

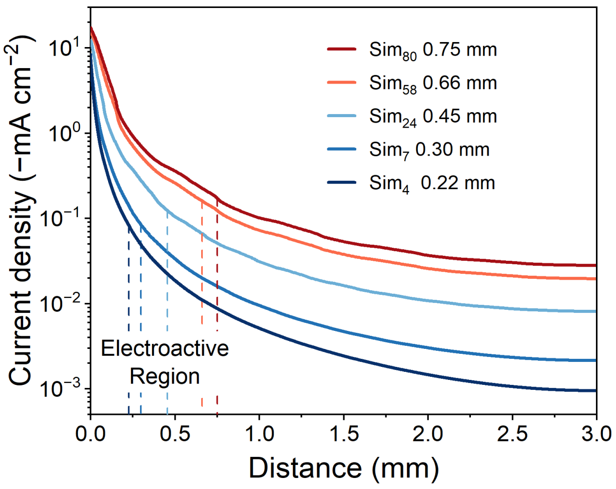

Supplementary Fig. 24. Interface current density distribution on EM channels with different pore diameters at an HRT_{geom} of 14 s. The left side of the dashed line represents the electroactive region.

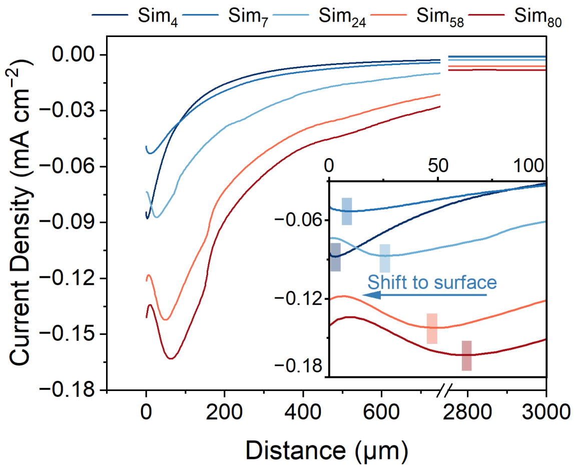

Supplementary Fig. 25. The simulated nitrate reduction current distribution at an HRT_{geom} of 91 s. The inset image presents an enlarged view of the current distribution profile, illustrating that the peak current shifts closer to the surface as the pore size decreases.

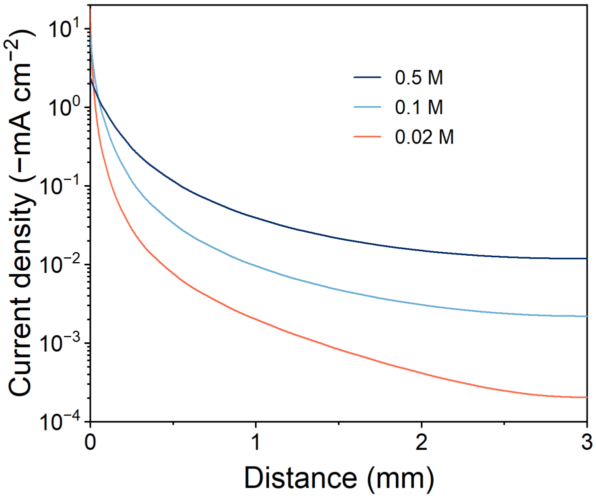

Supplementary Fig. 26. Simulation of current distribution in Sim_7 at different electrolyte concentrations.

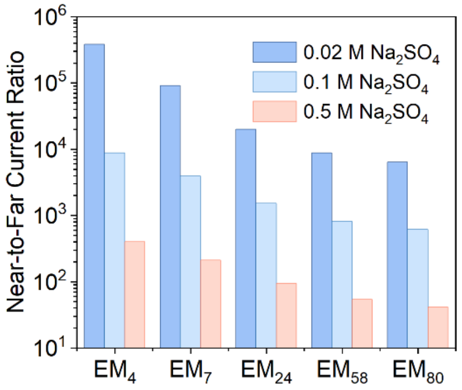

Supplementary Fig. 27. Ratios of current at the nearest end to that at the farthest end on EM channels with different pore diameters and different electrolyte concentrations.

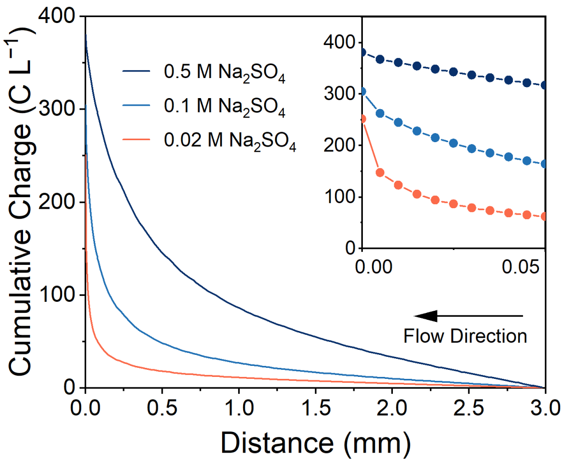

Supplementary Fig. 28. Cumulative charge of nitrate reduction on Sim_7 at different concentrations of electrolyte at an HRT_{geom} of 14 s. The inset image shows the enlarged cumulative charge distribution.

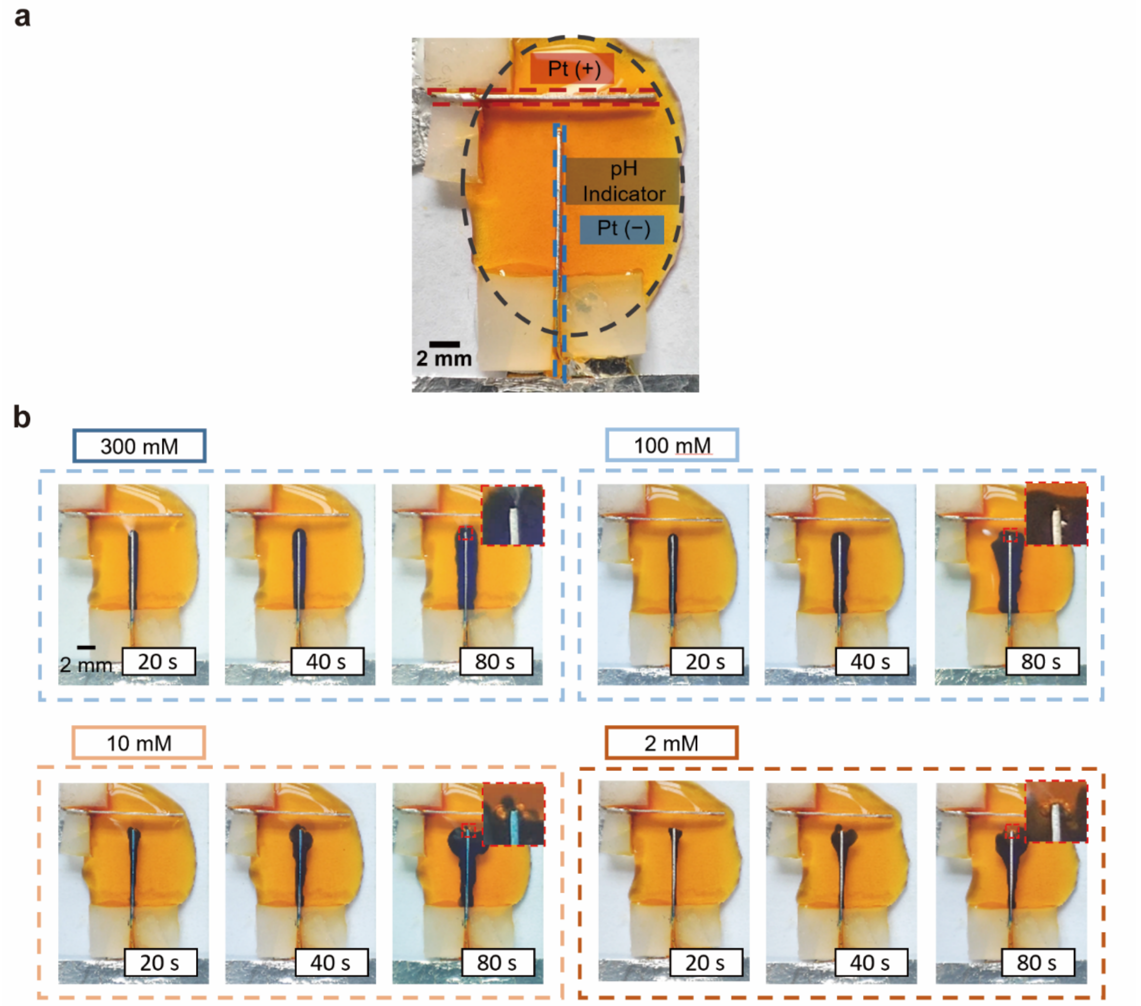

Supplementary Fig. 29. Optical observation of current density distribution on the Pt electrode surface across electrolyte concentrations ranging from 2 mM to 300mM. (**a**) Photograph of electrode configuration. (**b**) Evolution of pH distribution at electrode interface. Bromothymol blue was employed as a pH probe and the same setup as in the fluorescence observation experiment was used. Current: 0.6 mA.

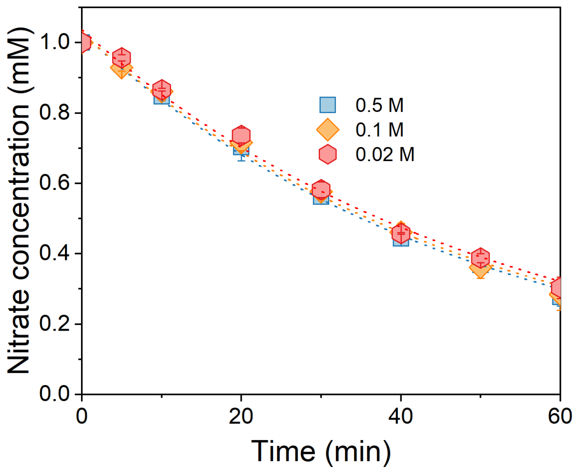

Supplementary Fig. 30. Removal of nitrate on EM_7 at different concentrations of electrolyte in the flow-by mode. The dotted lines represent the pseudo-first-order kinetic regression curves ($R^2 > 0.99$). Error bars represent the standard deviation from at least two independent tests.

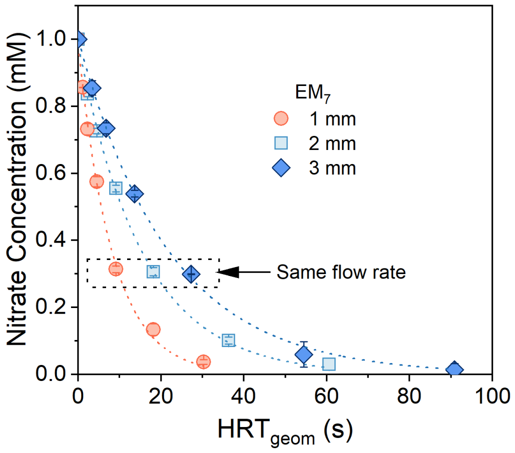

Supplementary Fig. 31. Nitrate removal performance on EM_7 with different thicknesses as a function of HRT_{geom}. The dotted lines represent the pseudo-first-order kinetic regression curves ($R^2 >$ 0.99). Error bars represent the standard deviation from at least two independent tests.

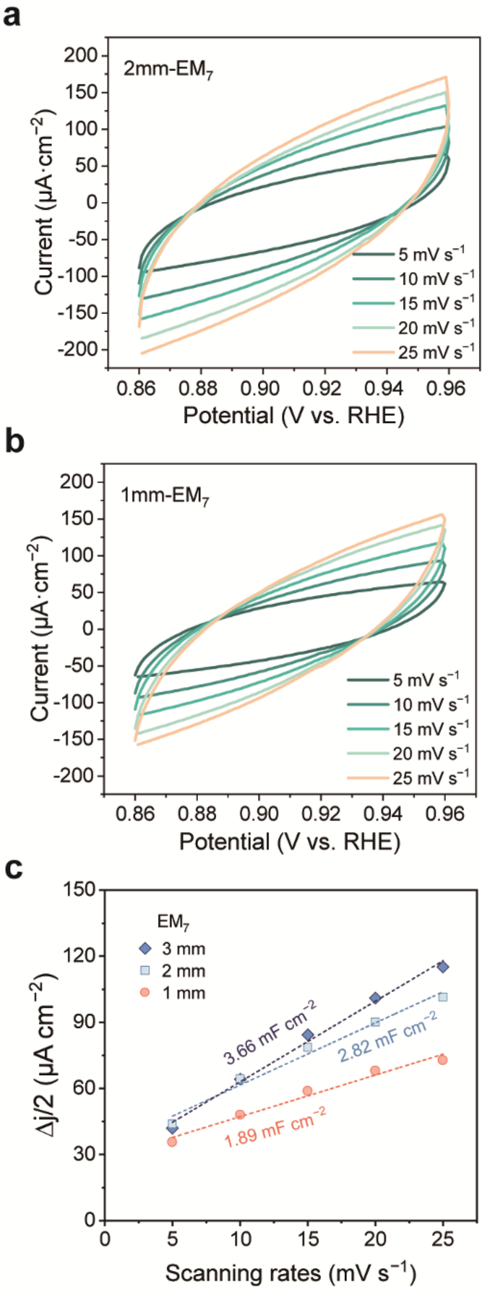

Supplementary Fig. 32. Cyclic voltammetry (CV) curves of EMs with different pore sizes and the corresponding electrical double-layer capacitance (C_{dl}). Panels correspond to (**a**) 2mm-EM_7, (**b**) 1mm-EM_7, and (**c**) C_{dl} of EM_7 with different thicknesses by plotting current variation against the scan rate to fit a linear regression. C_{dl} values were measured at the potential of 0.91 V vs. RHE. Electrolyte: 0.1 M Na_2SO_4.

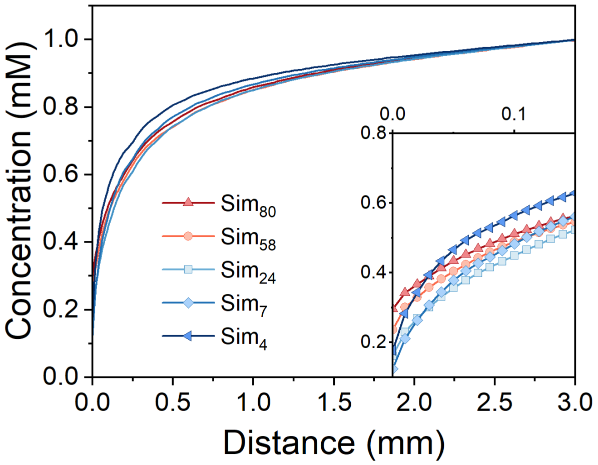

Supplementary Fig. 33. Simulated cross-sectional average nitrate concentration on EMs with different pore sizes at an HRT_{geom} of 55 s. The inset image shows the enlarged view of the nitrate concentration in the region near the pore opening.

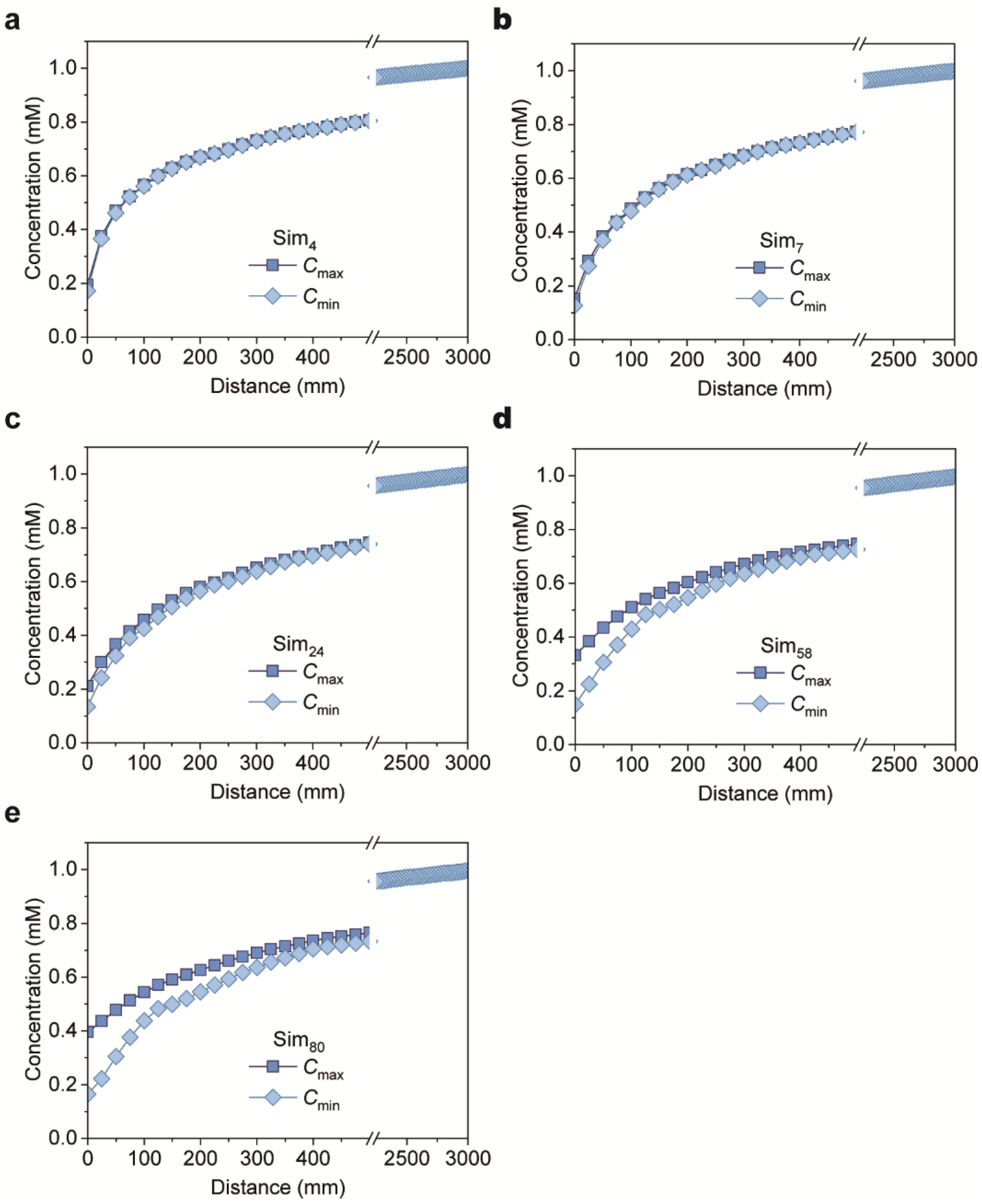

Supplementary Fig. 34. Nitrate concentration along the flow direction in Sims. Panels correspond to results of (**a**) Sim_4, (**b**) Sim_7, (**c**) Sim_{24}, (**d**) Sim_{58}, and (**e**) Sim_{80}. C_{min} and C_{max} represent the lowest and highest value of nitrate concentration in the cross-section, respectively.

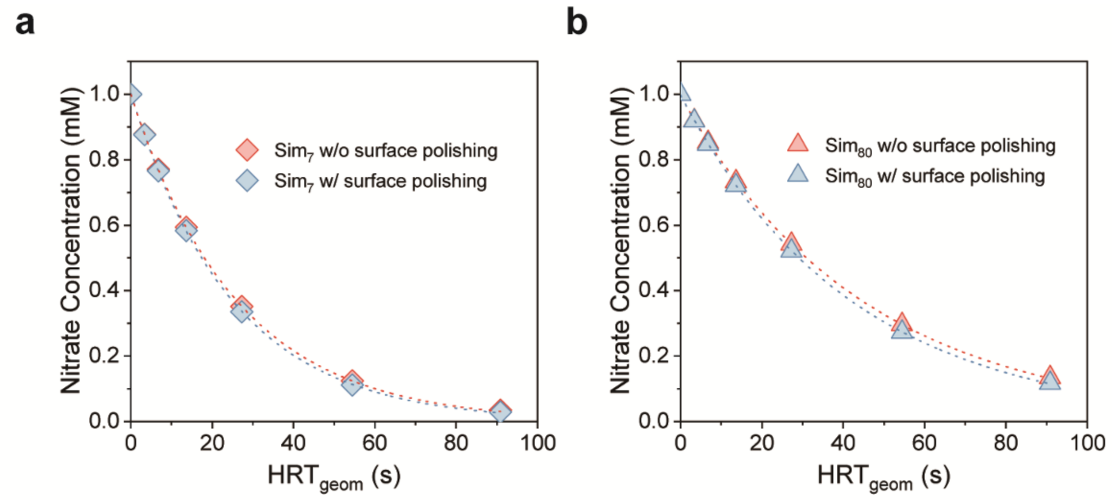

Supplementary Fig. 35. Simulated nitrate removal performance with surface polishing. Panels correspond to results of (**a**) Sim_7 and (**b**) Sim_{80}. The dotted lines represent the pseudo-first-order kinetic regression curves ($R^2 > 0.99$). Polishing refers to setting the exterior surface of the model to be non-catalytic. Electrolyte: 0.1 M Na_2SO_4.

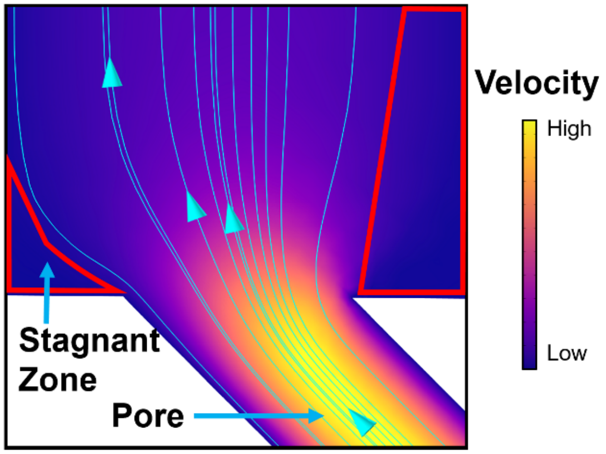

Supplementary Fig. 36. Schematic of the flow field on the upper surface of EMs.

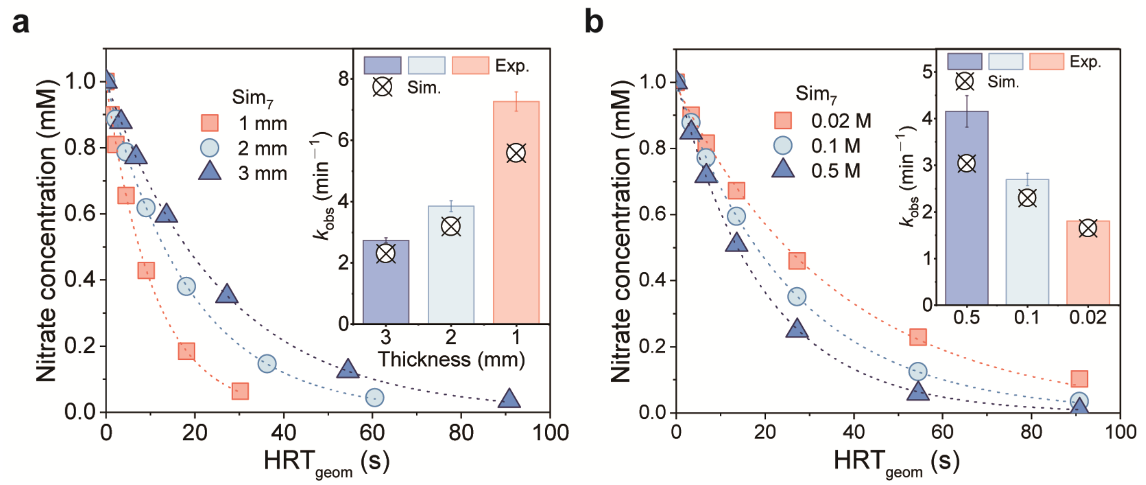

Supplementary Fig. 37. Simulated nitrate reduction performance on EM_7. Panels correspond to results at (**a**) different electrode thickness and (**b**) different concentrations of electrolyte. The inset image shows the corresponding pseudo-first-order kinetic constants. The dotted lines represent the pseudo-first-order kinetic regression curves ($R^2 > 0.99$). Error bars represent the standard deviation from at least two independent tests.

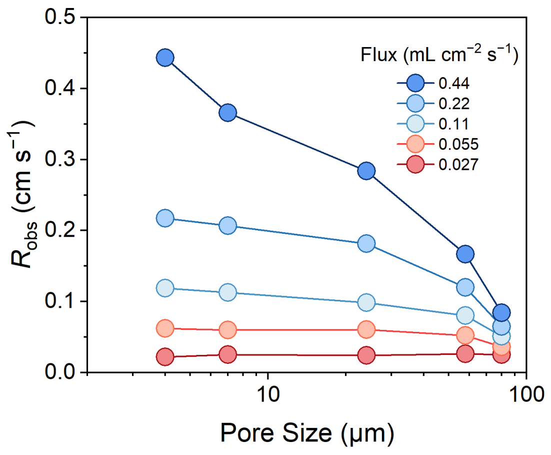

Supplementary Fig. 38. Mass transfer rate on EMs at different fluxes.

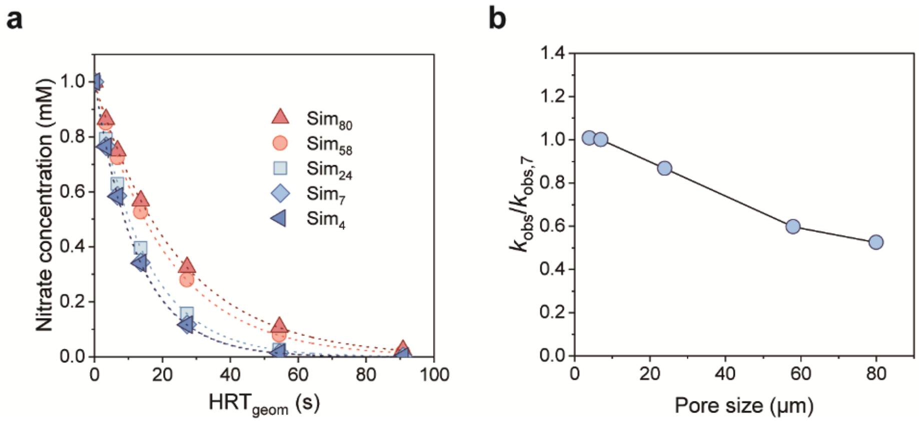

Supplementary Fig. 39. The simulation of EM channels with uniform current distribution. (**a**) The simulated nitrate removal performance when all model channels have a uniform current distribution. The dotted lines represent the pseudo-first-order kinetic regression curves (R^2 > 0.99). (**b**) The corresponding pseudo-first-order kinetic constants of nitrate reduction on Sims. To exclude any possible impact of nonuniform current distribution, we employed a first-order reaction model to represent nitrate reduction on the pore surface. The kinetic constant of the surface reaction was set to be proportional to the average local current density and kept uniform throughout the simulated channel.

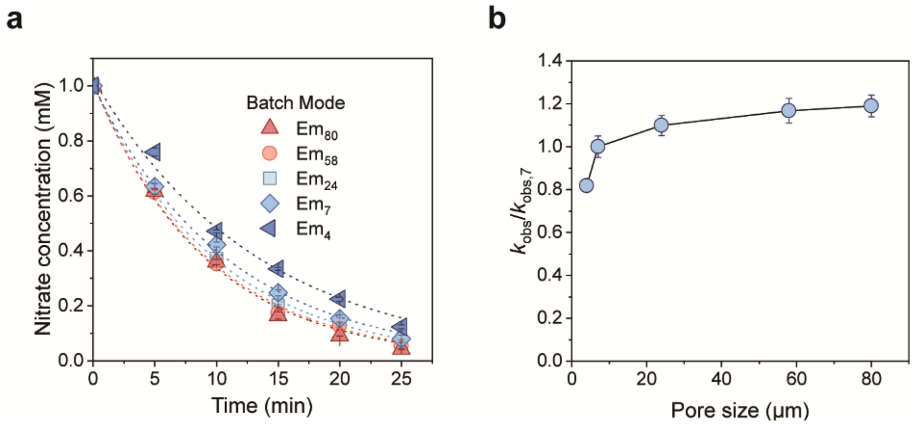

Supplementary Fig. 40. The performance of EMs with the same mass transfer rate at a batch mode. (**a**) The nitrate removal performance on EMs with the same mass transfer rate at a batch mode. The dotted lines represent the pseudo-first-order kinetic regression curves (R^2 > 0.98). (**b**) The corresponding pseudo-first-order kinetic constants of EMs. The experiments were carried out at batch mode (recirculating permeated water to the reservoir for continuous feeding) with a total liquid volume of 20 mL. The flux of each EM was adjusted to ensure they have the same mass transfer rate (0.07 cm s^{-1}). The flux of EM_{80}, EM_{58}, EM_{24}, EM_7, and EM_4 was 0.30, 0.10, 0.08, 0.07, and 0.07 mL cm^{-2} s^{-1}, respectively, according to Supplementary Figure.13. Error bars represent the standard deviation from at least two independent tests.

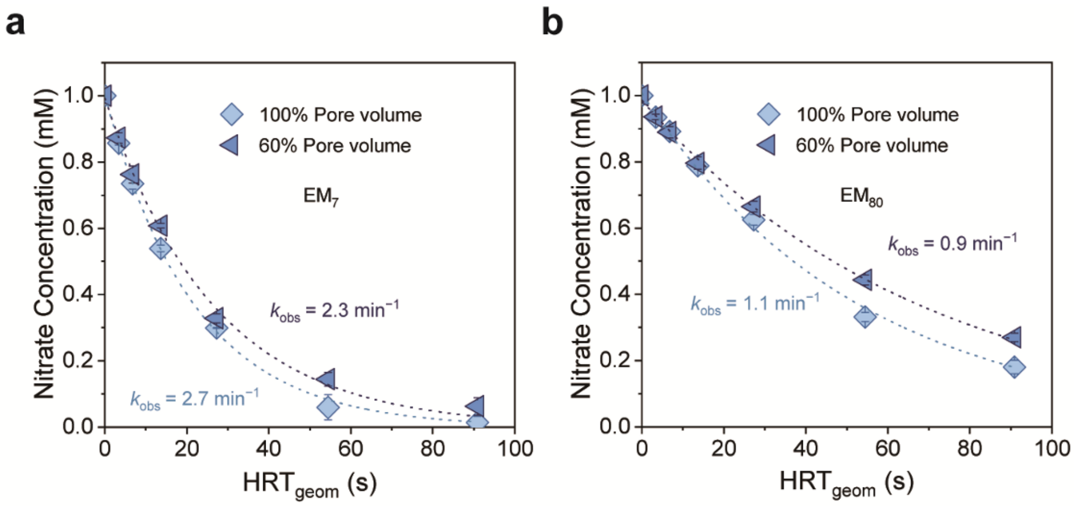

Supplementary Fig. 41. Catalytic performance of EMs with a 40% reduction in porosity. Panels correspond to results on (**a**) EM_7 and (**b**) EM_{80}. The dotted lines represent the pseudo-first-order kinetic regression curves (R^2 > 0.98). Error bars represent the standard deviation from at least two independent tests. Current density: 39.3 mA cm^{-2}.

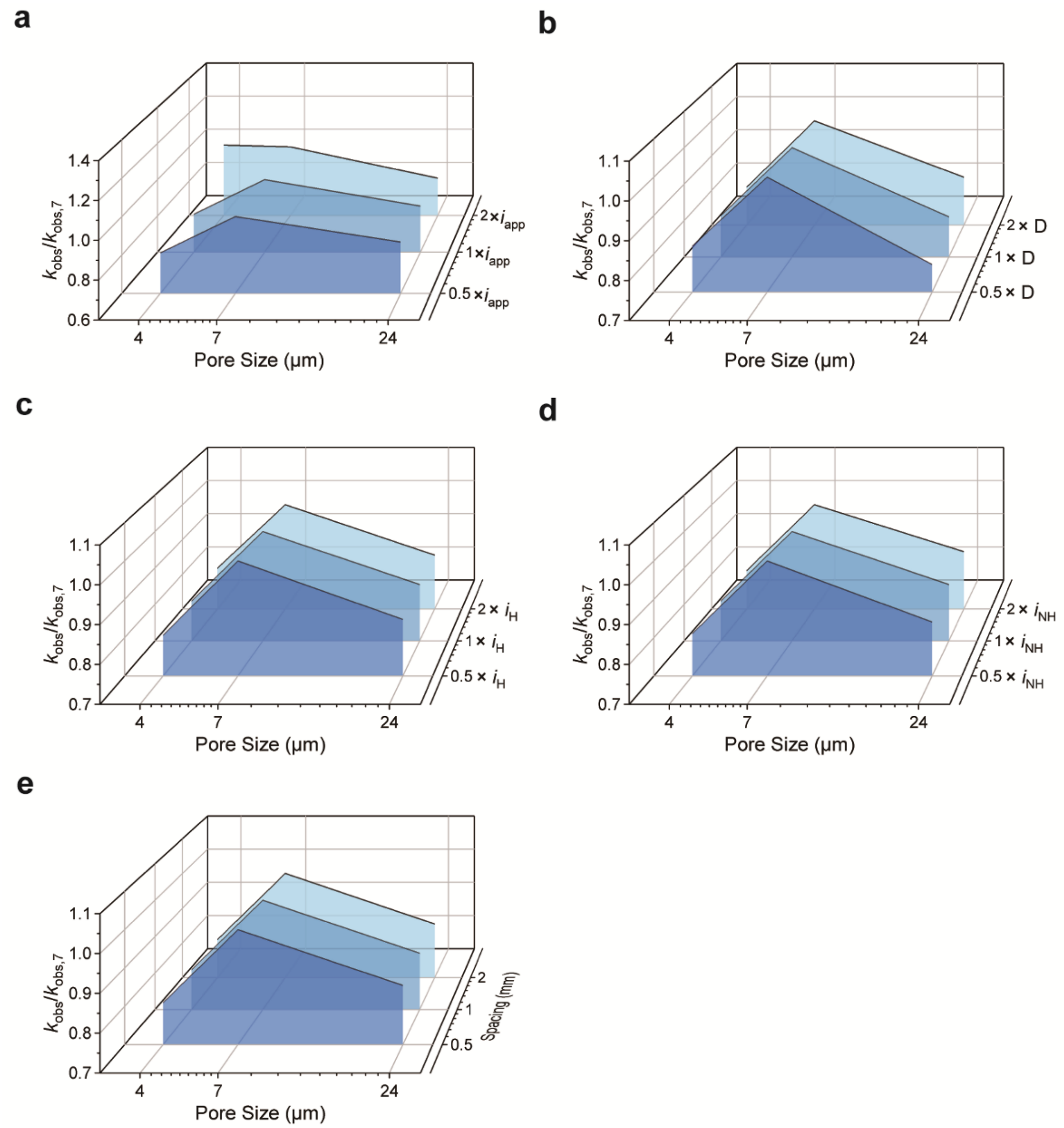

Supplementary Fig. 42. Simulated reaction kinetics of Sim_4, Sim_7, and Sim_{24} under varied conditions. The panels show the results under variations in (**a**) applied current, (**b**) reactant diffusion coefficient, (**c**) hydrogen evolution reaction exchange current density, (**d**) nitrate reduction exchange current density, and (**e**) electrode spacing. The y-axis represents the ratio of k_{obs} of sim_4 or sim_{24} to the k_{obs} of Sim_7 under an identical condition. $D = 1 \times 10^{-9}$ m^2 s^{-1}.

Supplementary Table 1. The surface area and porosity of EMs.

	BET Area [m^2 g^{-1}]	Mercury Intrusion Area[a] [m^2 g^{-1}]	C_{dl} [mF cm^{-2}]	Porosity[b] [%]
EM_4	10.90	3.23	4.44	10
EM_7	6.80	1.92	3.66	16
EM_{24}	3.47	1.76	2.85	23
EM_{58}	2.30	0.75	2.16	31
EM_{80}	2.08	0.53	1.60	35

[a]The mercury intrusion area is obtained from the cumulative specific surface area when the pore size is greater than 21 nm.

[b]The porosity of EMs was determined by Archimedes' Drainage Method.

Supplementary Table 2. The operating potential of the EMs.[a]

	Potential [V_{RHE}]
EM_4	−0.220 ± 0.003
EM_7	−0.212 ± 0.006
EM_{24}	−0.236 ± 0.003
EM_{58}	−0.238 ± 0.004
EM_{80}	−0.249 ± 0.004

[a]The potential was measured under conditions of 0.1 M Na_2SO_4 with 1 mM $NaNO_3$, at an $HRT_{\mathbf{geom}}$ of 7 s.

Supplementary Table 3. Detailed comparison of nitrate reduction current.[a]

Catalyst	Electrolyte	Potential [V_{RHE}]	Current density [mA cm^{-2}]	Ref
Ni_1Cu-SAA	0.5 M K_2SO_4<br>200 ppm NO_3^--N	−0.2	−20	17
IrNiCu@Cu-20	0.1 M KOH<br>0.1 M KNO_3	−0.1	−82	18
Co NC/Graphene	1 M KOH<br>1 M KNO_3	−0.2	−122	19
CoP-CNS	1 M OH^{-1}<br>1 M NO_3^-	−0.2	−102	20
Pd-CuO-200	1 M KOH<br>0.1 M KNO_3	−0.2, −0.1	−183, −139	21
Cu-NBs-100	1 M KOH<br>0.1 M KNO_3	−0.2	−350	22
Fe/Cu-HNG	1 M KOH<br>0.1 M KNO_3	−0.2	−15	23
$Cu_{50}Ni_{50}$	1 M KOH<br>0.1 M KNO_3	−0.1	−10	24

[a]All data used for comparison are derived from LSV measurements.

Supplementary Table 4. Energy consumption for nitrate removal on EMs.[a]

	Cell Voltage [V]	k_{obs} [min^{-1}]	$HRT_{geom,80\%}$[b] [s]	Q [10^{-4} m^3 h^{-1}]	ΔP[c] [kPa]	$E_{electrical}$[d] [kWh g-N^{-1}]	$E_{pumping}$[e] [kWh g-N^{-1}]	Energy Consumption [kWh g-N^{-1}]
EM_4	3.4	2.10	45	0.61	9.8	0.4987	0.0045	0.5032
EM_7	3.4	2.74	35	0.78	6.0	0.3895	0.0028	0.3923
EM_{24}	3.5	1.86	52	0.53	3.0	0.5906	0.0014	0.5920
EM_{58}	3.5	1.38	70	0.39	0.8	0.7961	0.0004	0.7964
EM_{80}	3.5	1.14	85	0.32	0.7	0.9636	0.0003	0.9640

[a]The energy consumption was calculated according to the methods described in the Supplementary Text. The concentration of nitrate is 1 mM.

[b]$HRT_{geom,80\%}$ refers to the time required to reach 80% nitrate removal.

[c]ΔP refers to the transmembrane pressure and is determined from Supplementary Fig. 18.

[d]$E_{electrical}$ refers to the electrical energy.

[e]$E_{pumping}$ refers to pumping energy.

Supplementary Table 5. Estimated flow velocity and *Re* of EMs.

	Highest flow velocity[a] [cm s^{-1}]	*Re*
EM_4	0.88	0.04
EM_7	0.55	0.04
EM_{24}	0.38	0.09
EM_{58}	0.28	0.16
EM_{80}	0.25	0.20

[a]The highest flow velocity in the pores was calculated based on the highest flux and porosity observed in the experiments.

Supplementary Table 6. Parameters and values used in the simulation.

Parameters	Value
Hydrogen evolution potential (E_H)[25]	0 [V]
Exchange current density for hydrogen evolution (i_H)[a]	1×10^{-2} [A m^{-2}]
Charge transfer coefficient for hydrogen evolution (α_H)[26] [a]	0.75
Nitrate reduction potential (E_{NH})[27]	0.69 [V]
Exchange current density for nitrate reduction (i_{NH})[a]	$5.7\times10^{-10}C_O^{*(1-\alpha)}C_R^{*\alpha}$ [A m^{-2}]
Charge transfer coefficient for nitrate reduction (α_{NH})[a]	0.6
Current of one simulated pore (i_{app})[b]	-5.6×10^{-6}, -3.4×10^{-6}, -7.7×10^{-7}, -1.0×10^{-7}, -5×10^{-8} [A]
Inlet concentration[c]	1×10^{-4}, 1×10^{-3}, 1×10^{-2} [M]
HRT$_{\mathbf{geom}}$ for nitrate reduction[c]	6.8 – 181.7 [s]
Electrolyte conductivity[c]	0.256, 1.28, 6.4 [S m^{-1}]
Diffusion coefficient[d]	1×10^{-9} [m^2 s^{-1}]
Tortuosity of pore model (τ)[e]	1.4

[a] Due to the three-dimensional structure of the EMs, these values were optimized in the simulation. The catalytic performance at different currents, fluxes, electrolyte concentrations and electrode thicknesses was also simulated to check the validity of the fitted model parameters. The influence of these optimized parameters on the simulation results is discussed in the Discussion section and Supplementary Fig 42.

[b] Current of EM_{80}, EM_{58}, EM_{24}, EM_7, and EM_4 in one simulated pore was determined according to Supplementary Equation (5), respectively.

[c] These values were determined by the experimental operating conditions.

[d] The diffusion coefficient of nitrate ions is around 1.5×10^{-9} m^2 s^{-1} [28]; however, since the model did not incorporate factors such as pore connectivity or bubble-induced mass transfer effects, a value of 1×10^{-9} m^2 s^{-1} was adopted in the simulation. The influence of this parameter on the simulation results is discussed in the Discussion section and Supplementary Fig. 42.

[e] The tortuosity of porous titanium foam ranges from 1.2 to 1.8[2], and a value of 1.4 was selected for the simulation.

Supplementary References

1 Khalid, Y. S., Misal, S. N., Mehraeen, S. & Chaplin, B. P. Reactive-transport modeling of electrochemical oxidation of perfluoroalkyl substances in porous flow-through electrodes. *ACS ES. T. Eng.* **2**, 713–725, (2022).

2 Chen-Wiegart, Y.-c. K. *et al.* 3D morphological evolution of porous titanium by x-ray micro- and nano-tomography. *J. Mater. Res.* **28**, 2444–2452, (2013).

3 Beck, V. A. *et al.* Inertially enhanced mass transport using 3D-printed porous flow-through electrodes with periodic lattice structures. *Proc. Natl. Acad. Sci. U. S. A.* **118**, e2025562118, (2021).

4 Boomsma, K., Poulikakos, D. & Ventikos, Y. Simulations of flow through open cell metal foams using an idealized periodic cell structure. *Int. J. Heat Fluid Flow* **24**, 825-834, (2003).

5 Donaghue, A. & Chaplin, B. P. Effect of select organic compounds on perchlorate formation at boron-doped diamond film anodes. *Environ. Sci. Technol.* **47**, 12391–12399, (2013).

6 Park, H. *et al.* In operando visualization of redox flow battery in membrane-free microfluidic platform. *Proc. Natl. Acad. Sci. U. S. A.* **119**, e2114947119, (2022).

7 Trainham, J. A. & j, N. A flow-through porous electrode model: Application to metal-ion removal from dilute streams. *J. Electrochem. Soc.* **124**, 1528-1540, (1977).

8 Trainham, J. A. & j, N. The effect of electrode placement and finite matrix conductivity on the performance of flow-through porous electrodes. *J. Electrochem. Soc.* **125**, 58–67, (1978).

9 Gu, Z., Zhang, Z., Ni, N., Hu, C. & Qu, J. Simultaneous phenol removal and resource recovery from phenolic wastewater by electrocatalytic hydrogenation. *Environ. Sci. Technol.* **56**, 4356–4366, (2022).

10 Ni, N. *et al.* Electrocatalytic deep hydrogenation of 4-chlorophenol into cyclohexanol on microchannel-enhanced Ru/TiO_2 for wastewater detoxification and simultaneous resource recovery. *J. Environ. Chem. Eng.* **11**, 109709, (2023).

11 Doherty, T., Sunderland, J. G., Roberts, E. P. L. & Pickett, D. J. An improved model of potential and current distribution within a flow-through porous electrode. *Electrochim. Acta* **41**, 519-526, (1996).

12 Bard, A. J. & Faulkner, L. R. *Electrochemical Methods: Fundamentals and Applications*. 2nd edn, (Wiley, 2001).

13 Gu, Z. *et al.* Interface-modulated nanojunction and microfluidic platform for photoelectrocatalytic chemicals upgrading. *Appl. Catal. B.* **282**, 119541, (2021).

14 Wang, L. *et al.* Blue TiO_2 nanotube electrocatalytic membrane electrode for efficiency electrochemical degradation of organic pollutants. *Chemosphere* **306**, 135628, (2022).

15 Yao, Y. *et al.* Phase change on stainless-steel mesh for promoting sulfate radical formation via peroxymonosulfate oxidation. *Appl. Catal. B.* **278**, 119333, (2020).

16 Lim, J., Yang, Y. & Hoffmann, M. R. Activation of Peroxymonosulfate by Oxygen Vacancies-Enriched Cobalt-Doped Black TiO_2 Nanotubes for the Removal of Organic Pollutants. *Environ. Sci. Technol.* **53**, 6972–6980, (2019).

17 Cai, J. *et al.* Electrocatalytic nitrate-to-ammonia conversion with ~100% Faradaic efficiency via single-atom alloying. *Appl. Catal. B.* **316**, 121683, (2022).

18 Xiong, Y. *et al.* Regulating the electrochemical nitrate reduction performance with controllable distribution of unconventional phase copper on alloy nanostructures. *Adv. Mater.* **36**, 2407889, (2024).

19 Kani, N. C. *et al.* Electrochemical reduction of nitrates on CoO nanoclusters-functionalized graphene with highest mass activity and nearly 100% selectivity to ammonia. *Adv. Energy Mater.* **13**, 2204236, (2023).

20 Fan, K. *et al.* Active hydrogen boosts electrochemical nitrate reduction to ammonia. *Nat. Commun.* **13**, 7958, (2022).

21 Liu, Y. *et al.* Shear-strained Pd single-atom electrocatalysts for nitrate reduction to ammonia. *Angew. Chem. Int. Ed. Engl.*, e202411396, (2024).

22 Hu, Q. *et al.* Reaction intermediate-mediated electrocatalyst synthesis favors specified facet and defect exposure for efficient nitrate–ammonia conversion. *Energy Environ. Sci.* **14**, 4989-4997, (2021).

23 Zhang, S. *et al.* Fe/Cu diatomic catalysts for electrochemical nitrate reduction to ammonia. *Nat. Commun.* **14**, 3634, (2023).

24 Lou, Y.-Y. *et al.* Phase-dependent electrocatalytic nitrate reduction to ammonia on Janus Cu@Ni tandem catalyst. *ACS Catal.* **14**, 5098-5108, (2024).

25 Zuo, K. *et al.* Electrified water treatment: fundamentals and roles of electrode materials. *Nature Reviews Materials* **8**, 472-490, (2023).

26 Guidelli, R. *et al.* Defining the transfer coefficient in electrochemistry: An assessment (IUPAC Technical Report). *Pure Appl. Chem.* **86**, 245-258, (2014).

27 Chen, F. Y. *et al.* Efficient conversion of low-concentration nitrate sources into ammonia on a Ru-dispersed Cu nanowire electrocatalyst. *Nat. Nanotechnol.* **17**, 759–767, (2022).

28 Yeh, H. & Wills, G. Diffusion coefficient of sodium nitrate in aqueous solution at 25. deg. as a function of concentration from 0.1 to 1.0 M. *J. Chem. Eng. Data* **15**, 187-189, (1970).
