# Supplementary material for: Confinement-enhanced valorization of contaminants in electrified hydrogenation membranes for water purification
Source: Nat Commun. 2025 Oct 27;16:9443. doi: 10.1038/s41467-025-64482-z (PMC12559343; doi:10.1038/s41467-025-64482-z)
Supplement: Supplementary file 2 — Description of Additional Supplementary File [file 41467_2025_64482_MOESM2_ESM.pdf]

Description of additional supplementary file

1. File Name: **Supplementary Movie 1:**

Description: Current distribution via fluorescent OH^{-} probe

2. File Name: **Supplementary Movie 2:**

Description: Current distribution under different electrolyte concentrations via pH probe
